# Intracellular Uropathogenic *E*. *coli* Exploits Host Rab35 for Iron Acquisition and Survival within Urinary Bladder Cells

**DOI:** 10.1371/journal.ppat.1005083

**Published:** 2015-08-06

**Authors:** Neha Dikshit, Pradeep Bist, Shannon N. Fenlon, Niyas Kudukkil Pulloor, Christelle En Lin Chua, Marci A. Scidmore, Jason A. Carlyon, Bor Luen Tang, Swaine L. Chen, Bindu Sukumaran

**Affiliations:** 1 Program in Emerging Infectious Diseases, Duke-NUS Graduate Medical School, Singapore; 2 Infectious Diseases Group, Genome Institute of Singapore, Singapore; 3 Faculty of Medicine and Institute for Life Sciences, University of Southampton, Southampton, United Kingdom; 4 Department of Biochemistry, Yong Loo Lin School of Medicine, National University of Singapore, Singapore; 5 Department of Microbiology and Immunology, College of Veterinary Medicine, Cornell University, Ithaca, New York, United States of America; 6 Department of Microbiology and Immunology, Virginia Commonwealth University School of Medicine, Richmond, Virginia, United States of America; 7 Department of Medicine, Division of Infectious Diseases, Yong Loo Lin School of Medicine, National University of Singapore, Singapore; University of Virginia School of Medicine, UNITED STATES

## Abstract

Recurrent urinary tract infections (UTIs) caused by uropathogenic *E*. *coli* (UPEC) are common and morbid infections with limited therapeutic options. Previous studies have demonstrated that persistent intracellular infection of bladder epithelial cells (BEC) by UPEC contributes to recurrent UTI in mouse models of infection. However, the mechanisms employed by UPEC to survive within BEC are incompletely understood. In this study we aimed to understand the role of host vesicular trafficking proteins in the intracellular survival of UPEC. Using a cell culture model of intracellular UPEC infection, we found that the small GTPase Rab35 facilitates UPEC survival in UPEC-containing vacuoles (UCV) within BEC. Rab35 plays a role in endosomal recycling of transferrin receptor (TfR), the key protein responsible for transferrin–mediated cellular iron uptake. UPEC enhance the expression of both Rab35 and TfR and recruit these proteins to the UCV, thereby supplying UPEC with the essential nutrient iron. Accordingly, Rab35 or TfR depleted cells showed significantly lower intracellular iron levels and reduced ability to support UPEC survival. In the absence of Rab35, UPEC are preferentially trafficked to degradative lysosomes and killed. Furthermore, in an *in vivo* murine model of persistent intracellular infection, Rab35 also colocalizes with intracellular UPEC. We propose a model in which UPEC subverts two different vesicular trafficking pathways (endosomal recycling and degradative lysosomal fusion) by modulating Rab35, thereby simultaneously enhancing iron acquisition and avoiding lysosomal degradation of the UCV within bladder epithelial cells. Our findings reveal a novel survival mechanism of intracellular UPEC and suggest a potential avenue for therapeutic intervention against recurrent UTI.

## Author Summary

Urinary tract infections (UTIs) are common and costly infectious diseases, affecting half of all women. Many women suffer from recurrent UTIs, for which no effective therapy currently exists. Intracellular persistence within bladder epithelial cells (BEC) by uropathogenic *E*. *coli* (UPEC) contributes to recurrent UTI in mouse models of infection. In the current study, we specifically asked whether and how UPEC co-opt any of the host proteins regulating vesicular trafficking for intracellular infection. Our study demonstrates a novel mechanism by which UPEC exploit a host endocytic recycling pathway protein (Rab35) to acquire the critical nutrient iron and to prevent lysosomal degradation, thereby promoting intracellular survival within BEC. The results of this study may highlight new avenues for therapeutic intervention in recurrent UTI. In addition, knowledge gained from this study can also be extended to understand the general principles by which other intracellular bacterial pathogens acquire essential nutrients, leading to additional strategies to combat these infectious diseases.

## Introduction

Urinary tract infections (UTIs) are one of the most common bacterial infections in humans, affecting at least 50% of women at some point in their lifetime. UTIs constitute significant morbidity and economic burden, accounting for more than 1 million hospitalizations and $2.4 billion in medical expenses in the USA alone annually [1,2]. Most (>80%) UTIs are caused by *Escherichia coli*, thus the term uropathogenic *E*. *coli* (UPEC) [3]. After an initial infection, 25% of patients suffer a recurrence within 6 months, with 68% of these UTIs apparently caused by the original strain, despite appropriate antibiotic therapy [4,5]. Mouse models of UTI have been used by many groups to elucidate mechanisms underlying UPEC pathogenesis [6–8]. Experimentally infected mice also suffer episodes of recurrent UTI subsequent to clearance of bacteriuria following antibiotic therapy [9]. These recurrent infections are due to UPEC that persist within urinary bladder epithelial cells. UPEC have been described to form several types of intracellular populations *in vivo*, such as intracellular bacterial communities (IBCs) and quiescent intracellular reservoirs (QIRs), that contribute to phenotypic antibiotic resistance and evasion of the host immune response [10]. QIRs, in particular, cause persistent infections lasting for months in mice [11], while IBCs and other intracellular bacteria have been detected in the urine of both adult and pediatric human UTI patients [12–15]. Therefore, understanding the molecular mechanisms underlying the establishment of the intracellular bacterial reservoirs is critical for the development of efficient therapeutic strategies to control recurrent UTIs.

UPEC utilize type 1 pili, which carry the FimH adhesin at their distal tips, to specifically bind to mannosylated surface proteins (including uroplakins [16] and α3 and β1 integrins [17] on the surface of bladder epithelial cells [18]). Upon binding, UPEC are internalized into cyclic AMP- modulated Rab27b/CD63+ vesicles [19]. Internalization requires components of the host cell cytoskeleton and activation of Rho GTPases, various kinases, and other signaling intermediates [17,20–24]. Most of the internalized bacteria are expelled out of urothelial cells by Rab27b-mediated exocytosis [25]. *In vivo*, however, some bacteria escape their enclosing vesicle, enter the cytosol, and subsequently multiply to form biofilm-like IBCs that contain 10^4^−10^5^ bacteria each [26,27]. As a host countermeasure, infected epithelial cells undergo an apoptotic-like cell death, detaching from the urothelium to be expelled in the urine with the internalized bacteria [28]. However, this exposes deeper layers of the urothelium to infection, which is thought to lead to QIR formation. In contrast to IBCs, QIRs are smaller structures (6–8 bacteria), enclosed in host membranes (LAMP1, ATG16L1, and LC3- positive and CathepsinD negative), and found in immature epithelial cells [11,29]. However, the molecular profiles of these bacterial reservoirs and the interplay between UPEC and the host resulting in their formation are still in need of further characterization.

For successful intracellular survival, UPEC must obtain sufficient nutrients from the host cell. In mammals, iron is tightly bound to plasma and cellular proteins such as hemoglobin, transferrin, lactoferrin, or ferritin, which limit its availability to microbial pathogens, a phenomenon termed nutritional immunity [30]. In order to acquire iron, UPEC, like other bacteria, synthesize several siderophores (catecholates enterobactin and salmochelin, the hydroxamate aerobactin, and yersiniabactin, a mixed-type siderophore) and their cognate receptors [31]. These iron acquisition systems are important for virulence in mouse models of UTI and are among the most highly upregulated genes in both mouse [32,33] and human infections [34,35]. They are therefore being actively pursued as vaccine candidates [36–39]. With only one exception, all of these studies have focused on the expression of iron acquisition systems by extracellular bacteria. Notably, in the sole intracellular study [32], host cell transferrin receptor (TfR) levels increased within IBC-containing epithelial cells and epithelial cells near IBC-containing cells. Transferrin is the major iron carrier protein in the blood. Transferrin bound iron (Fe^3+^) is internalized from the circulation by the ubiquitously expressed TfR in almost all cells [40]. TfR trafficking is regulated in host cells by Rab GTPases [41]. Rab proteins are a subfamily of the Ras superfamily of GTPases that regulate various aspects of intracellular membrane trafficking [42]. Many intracellular pathogens that reside within host cell-derived vacuoles selectively recruit Rab proteins to facilitate trafficking of nutrient-rich vesicles to their vacuolar niches. Several pathogens also use Rab recruitment to prevent fusion of their vacuoles with degradative lysosomal compartments [43]. The connection between access to iron, transferrin, and Rab proteins was first noted in *Mycobacterium tuberculosis*, which associates with Rab11, a key regulator of the transferrin recycling pathway, to acquire iron [44]. Recent studies have identified another Rab protein, Rab35, a component of the rapid endocytic recycling pathway, that plays an important role in the recycling of endocytosed transferrin receptors (TfR) to the cell surface [45,46]. Upon internalization, the iron loaded transferrin-TfR complex is delivered to the early endosomes, where the Fe^3+^ dissociates from the transferrin. The receptor and transferrin are then recycled to the cell surface by Rab35 positive recycling endosomes to initiate a new round of iron uptake [47,48]. Inside the endosome, Fe^3+^ is reduced to Fe^2+^, which is subsequently released into the labile iron pool (LIP) in the cytosol, where it may be utilized or stored in complex with ferritin [49].

Similar to Rab11 recruitment by Mycobacteria, Rab35 has been shown to be recruited to the vacuoles of *Anaplasma phagocytophilum* [50], although its functional relevance in the intracellular persistence of pathogens has not yet been investigated. We hypothesized that Rab35 might play a role in iron acquisition during intracellular infection by UPEC. We found that UPEC infecting cultured bladder epithelial cells do indeed recruit Rab35 to their enclosing vesicles, structures we term the UPEC containing vacuoles (UCV). In a mouse model of persistent UPEC infection, UPEC within the uroepithelium also associates with Rab35. We found that Rab35 recruitment leads to increased TfR association with the UCV, which supports UPEC survival through the provision of iron. Finally, Rab35 recruitment serves a second function for UPEC survival by avoidance of UCV fusion with degradative lysosomes. Therefore, Rab35 recruitment is a key feature of the UPEC strategy for exploiting host vesicular trafficking during intracellular infection.

## Results

### Rab35 is recruited to the UPEC containing vacuole during intracellular infection of urinary bladder cells

**Fig 1:**
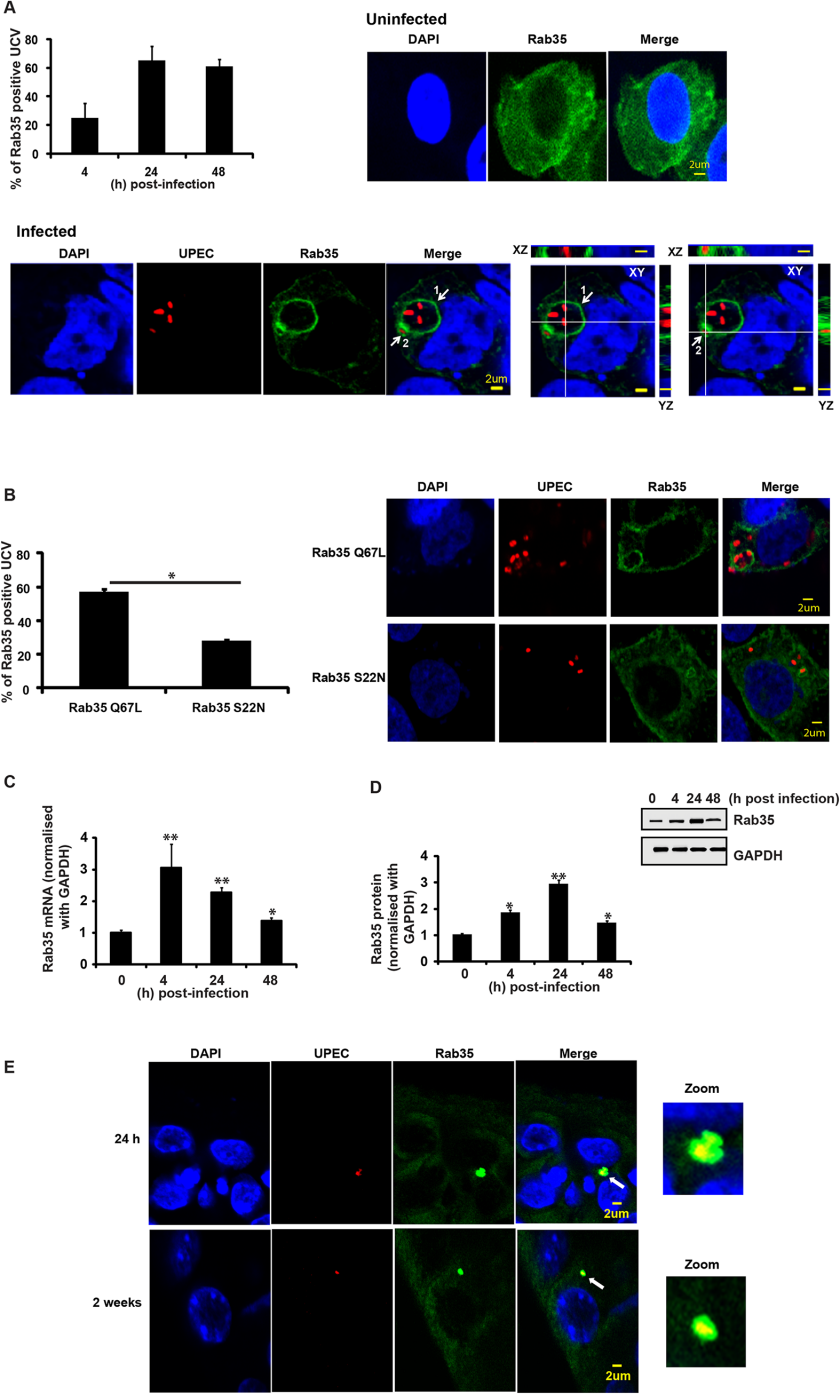
Rab35 protein localizes to UPEC-containing vacuole and is up regulated during UPEC infection. **A.** BEC cells were transfected with GFP-Rab35. At 24 h post transfection the cells were either left uninfected or infected (with RFP-UPEC (MOI 500) and the localization of Rab35 with UPEC-containing vacuole (UCV) was analyzed at 4, 24 and 48 h post-infection by confocal microscopy. UCV with complete rim-like demarcation of Rab35 (category 1, marked as 1 in the merged image) and UCV where Rab35 may not form a complete rim-like structure, but enriched at the periphery of UCV membrane (category 2, marked as 2 in the merged image) by visual determination were counted as the positive population for Rab35 positive UCV. Number of Rab35 positive UCV was divided by the total number of UCVs and is represented in the graph as % of Rab35 positive UCV. Representative z sections of the images at 24 h are shown. Also shown at the bottom right side are the orthogonal sections of bacteria in category 1 and category 2 UCVs in XZ and YZ plane. White lines represent regions in which XYZ sections were taken. Scale bar denotes 2μm. DAPI (blue), Rab35 (green), and UPEC (red). At least 100 UCVs were counted for each experiment. Experiments were repeated three times with similar results. **B**. BEC cells were transfected with Rab35 ∆Q67L or Rab35 ∆S22N as mentioned above. The cells were infected with RFP-UPEC and the localization of mutant Rab35 with UCV was analyzed at 24 h post infection by confocal microscopy and represented in the graph as % of Rab35 positive UCV (category 1 and 2) as mentioned above. A representative image at 24 h is shown in the right panel. **C** and **D**. BEC cells were infected with UPEC and Rab35 mRNA and protein expression was analyzed at 4, 24 and 48 h post infection by qRT-PCR (Fig **1C**) and Western blot (Fig **1D**) respectively. The qRT-PCR values are normalized with GAPDH. * represents p < .05, ** represents p < .01. All the values shown represent mean ± standard deviation of results from three independent experiments. Quantitation of the Western blots using the software ImageJ is shown (Fig **1D**, bottom panel). Relative densitometric data (Rab35 protein normalized with GAPDH) is shown. Values shown represent means ± standard deviations of results of three experiments. (* *p*<0.05; ** represents *p*<0.01 *vs* values at 0 h time point). **E**. C57BL/6 mice were infected transurethrally with UPEC (UTI89 strain). Mouse bladders were removed 24 h (top panels) and 2 weeks (bottom panel) post infection and the tissue sections were processed for immunofluorescence. The magnified image of association of Rab35 with UPEC is shown in the zoomed panel {(optical magnification (63x) and electronic zoom (2x)}. Green (Rab35) UPEC (red) and DAPI (blue). n = 4 sections/mouse bladder, n = 3 mice per experiment.

To identify host cell molecules and pathways utilized by UPEC for intracellular survival within BEC, we first focused on membrane trafficking pathway proteins. Rab GTPases are critical regulators of mammalian membrane trafficking pathways and many pathogenic bacteria are known to exploit these proteins for their intracellular survival within the host [43] by recruiting (or excluding) specific Rab proteins to bacteria containing compartments. We hypothesized that UPEC might subvert Rab GTPases for intracellular survival, possibly by recruiting specific Rab proteins to intracellular UPEC-containing compartments. To examine this, we initially focused on a subset of 15 human Rab GTPase proteins [50] and assessed their localization patterns during the UPEC intracellular infection, using the well-established bladder epithelial cell line 5637 (BEC5637) based infection model system [19]. BEC cells over-expressing GFP/EGFP-tagged Rab GTPases were infected with UPEC (CI5 strain) for various time intervals (4, 24 and 48 h). Based on previously reported data [11], we reasoned that intracellular bacterial levels at 4 h post-infection would represent the number of bacteria that had invaded the BEC or intracellular bacteria levels during the early stages of infection, while the number of intracellular bacteria at 24 and 48 h (or ≥ 24 h post-infection) post-infection would represent the surviving bacterial population (or bacterial levels during late stages of infection). In comparison to other tested Rab proteins (S1A Fig), Rab35 showed a rather striking degree of localization to UPEC-enriched vesicular structures, which we termed UPEC-containing vacuoles (UCV). BEC5637 cells over expressing GFP-tagged Rab35 protein were infected with UPEC for 4, 24 and 48 h. As shown in the graph (Fig 1A), a major fraction of Rab35 was found to associate with UCV at all the tested time points, as observed by confocal microscopy. There was a notable increase in the percentage of Rab35 localizing to the UCV membrane from 4 h to 24 h; but thereafter this value remained fairly constant. Representative confocal images of infected (24 h post-infection) and uninfected samples are also shown. Depending on the stage of UCV development, we observed three categories of associations of Rab35 with UCV/UPEC at the 24 h time point. (1) UCV with ~2–10 bacteria, showing a distinct rim-like demarcation of Rab35 enriched at the periphery of UCV membrane (Fig 1A, marked as 1 in the merged image); (2) UCV with ~1–2 bacteria, where Rab35 may not always outline a complete rim-like structure, but is nonetheless enriched at the periphery of UCV membrane (Fig 1A, marked as 2 in the merged image); (3) Single UPEC where an UCV is not very well developed, but the bacteria appear to be associated with cytoplasmic patches of Rab35 (S1B Fig, % seen = 30 ± 8). The first two categories were counted as the positive population (% seen = 64 ± 8) for Rab35 association with the UCV. On the other hand, UPEC-infected BEC5637 cells transfected with GFP showed diffuse green fluorescence throughout the cell with no specific GFP signal association with UCV (S1C Fig). Interestingly, comparison of infected and uninfected GFP-Rab35 expressing cells revealed that UPEC infection resulted in a distinct pattern of Rab35’s localization and membrane association within the infected cells (Fig 1A). While the uninfected cells showed a rather uniform distribution of Rab35 in the cytoplasm, UPEC infection induced a notable recruitment leading to increased presence of Rab35 around the UCVs. Rab35 recruitment was specific for UPEC as a commensal K12 *E*. *coli* strain that expresses type 1 pili (MG1655) was unable to recruit Rab35 (S1D Fig). In addition, we also found that heat-killed UPEC was unable to recruit Rab35 (S1E Fig). Rab35 is involved in the rapid endocytic recycling pathway and regulates membrane traffic via cyclical conversion between its active GTP-bound state and its inactive GDP-bound state [51]. We reasoned that if UPEC co-opts Rab35 to modulate a cellular process, it might preferentially associate with the GTP-bound, functionally active form of Rab35. Indeed, we observed that UPEC preferentially associated with the constitutively active Rab35 Q67L mutant (GTP locked) as compared to dominant negative Rab35 S22N (GDP bound) (2 fold difference, *p* = 0.03) (Fig 1B). This suggests that not only do UPEC recruit Rab35 to its vacuole, but it also possibly utilizes the transport pathways regulated by Rab35 for its survival. We also found an increase in the expression of Rab35 mRNA (2.2 fold at 24h, *p =* 0.008; Fig 1C) as well as protein (3 fold at 24h, *p =* 0.005; Fig 1D) with the progression of infection.

We also examined whether Rab35 is associated with intracellular UPEC during *in vivo* infection using a well-established murine UTI infection model [52]. Mice were infected with a cystitis isolate of UPEC, and bladders were removed at 24 h and 2 week time points, fixed, sectioned, and stained for UPEC and Rab35. We found that approximately 25% of the UPEC in bladder sections, found in small intracellular collections resembling QIRs, were associated with Rab35 (n = 4 sections/mouse bladder, n = 3 mice per experiment). An example of this association is shown in Fig 1E (top panels shows results from 24 h and bottom panel shows results from 2 weeks post-infection). As not all UPEC were colocalized with Rab35, we could conclude that the Rab35 staining was not due to nonspecific binding of the Rab35 antibody to UPEC (S1F Fig). Previous *in vivo* studies have shown that UPEC resides in a Lysosomal-associated membrane protein 1 (LAMP1, a late endosomal/early lysosomal marker) positive and Cathepsin D (late lysosomal marker) negative compartment in bladder cells during persistent infection (QIRs) [11]. We also observed that the Rab35-positive QIRs were also positive for LAMP1 (S2A Fig). Interestingly, similarly to ATG16L1 and LC3, which associate with both QIRs at 2 weeks post-infection and with IBCs at 6 h post-infection [29], we found that Rab35 also colocalized with immature IBCs at 6 h post-infection (S2B Fig).

### Rab35 plays a role in intracellular UPEC infection of urinary bladder epithelial cells

**Fig 2:**
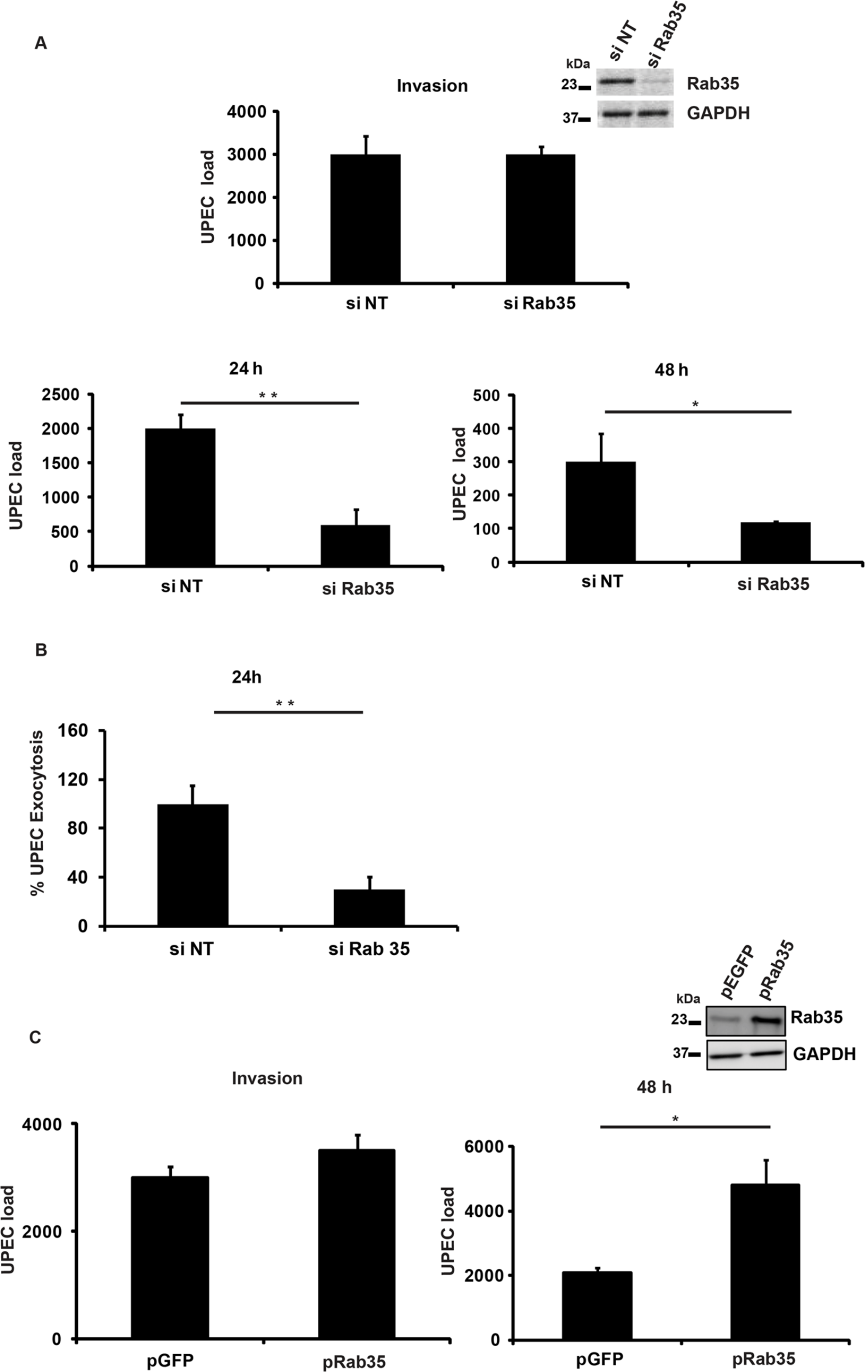
Rab35 is required for the survival of UPEC within BEC 5637. **A.** BEC5637 cells were transfected with 100nM each of si Rab35 or non-targeting siRNA (si NT). 48h following knockdown, the cells were infected with UPEC at MOI 500. Intracellular bacterial load at different time points {4 h (invasion), 24 h and 48 h, was determined by lysing the cells in 0.1% Triton X-100 and plating on LB-agar as described in Materials and Methods. Results are expressed as bacterial load/ 2x10^5^ cells. Immunoblotting was done to confirm the knock down efficiency of Rab35 siRNA (inset). **B.** Rab35 silencing does not enhance the efflux rate of UPEC from BEC-5637. BEC-5637 cells were transfected with 100nM each of si Rab35 or non-targeting siRNA (si NT). 48 h following knockdown, the cells were infected with UPEC at MOI 500. After gentamycin (100μg/ml) treatment, cells were washed in left in fresh culture medium containing 100mM methyl-D-mannopyranoside. At 24 h post infection, the culture medium was collected and plated for CFU counts as described in Materials and Methods. Results are expressed % exocytosis relative to siNT cells. **C.** Over expression of Rab35 protein leads to increase in bacterial load at 48 h post infection. BEC cells were transfected with GFP-Rab35 or control GFP vector for 24 h followed by UPEC infection (MOI 500) for another 48 h. Intracellular bacterial load was calculated as mentioned above. Results are expressed as bacterial load/ 2x10^5^ cells. Immunoblotting was done to confirm the expression levels of Rab35 (inset). * represents *p* < .05; ** represents *p*<0.01. Values shown represent mean ± standard deviation of results of three independent experiments.

Since we observed localization of Rab35 to UCV *in vitro* and to IBCs and QIRs *in vivo*, we next asked whether Rab35 played a functional role during UPEC infection of bladder epithelial cells. Using a short interfering RNA (siRNA) mediated gene-silencing approach, we knocked down the expression of Rab35 in BEC5637 cells and then infected them with UPEC. The ability of these cells to support UPEC infection was assessed at various time points by cfu enumeration. Gene knock down was confirmed by Western blot analysis (Fig 2A, inset). Rab35 silencing did not significantly affect the invasion/entry (4 h) of UPEC into bladder cells (Fig 2A). Remarkably, we found that silencing of *Rab35* led to a significant reduction in the intracellular bacterial load of BEC5637 cells at 24 and 48 h post infection (3.3 fold at 24 h, *p* = 0.01 and 2.5 fold at 48 h, *p* = 0.04) (Fig 2A). Infected BECs have the capacity to expel UPEC [19]. We also measured the levels of UPEC that are expelled from Rab35 silenced cells at 4 h and at 24 h post-infection. There was no change in the % of UPEC expelled from Rab35 silenced cells compared to control at 4 h post-infection (S2C Fig). At 24 h post-infection, there were reduced levels of UPEC expulsion from Rab35 silenced cells (Fig 2B). This experiment ruled out the possibility that the observed reduction in the intracellular UPEC load in Rab35 silenced cells is due to enhanced expulsion of bacteria; instead, the reduction appears to be due to reduced survival of intracellular UPEC. Consistent with the knockdown data, ectopic overexpression of Rab35 protein (GFP-Rab35) led to enhanced levels of UPEC (2.3-fold, *p* = 0.03) in bladder epithelial cells, as assessed at 48 h, although invasion of UPEC into BEC cells was not affected (Fig 2C). Expression levels of Rab35 were assessed by Western blot analysis (Fig 2C inset). These results demonstrate that Rab35 plays a role in the intracellular survival of UPEC in bladder epithelial cells.

### UPEC are targeted to a Cathepsin D-positive compartment in the absence of Rab35 expression

**Fig 3:**
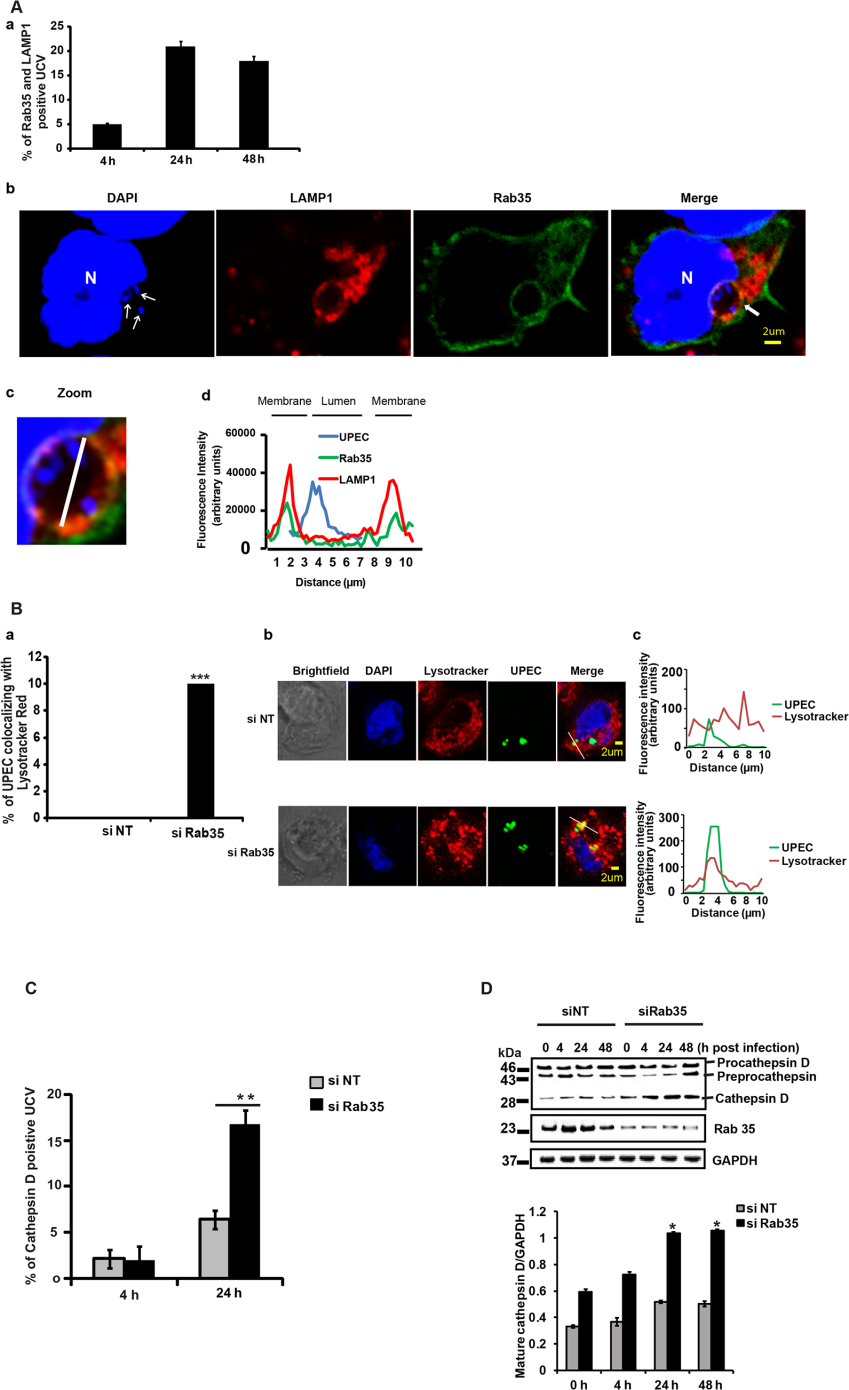
Loss of Rab35 targets UPEC to late lysosomal compartment. **A.** BEC-5637 cells were transfected with Rab35 expressing plasmid. 24 h later the cells were infected with UPEC. The cells were fixed at different stages of infection, permeabilised and stained for LAMP1 and were then analyzed by confocal microscopy. The results (panel a) are represented as % of UCV positive for both Rab35 and LAMP1. At least 100 UCV were counted for each experiment. A representative image is shown in panel b. DAPI {blue, host nuclei (N) or bacteria (UPEC)}, Rab35 (green), and LAMP1 (red). Arrows in the DAPI panel indicates UPEC. Zoomed panel (panel c) shows the magnified image of UCV (marked by arrows in the merged image) {(optical magnification (63x) and electronic zoom (2.5x)}. The graph (panel d) shows the quantification of the fluorescence intensity along the white line shown in the zoomed panel. Both LAMP1 and Rab35 co-localized at the UCV. **B.** si NT and si Rab35 cells were infected with GFP-UPEC. At 24 h post infection, the cells were stained with LysoTracker Red for 2 h; fixed and analyzed by confocal microscopy. Graph (panel a) is depicted as % bacteria co-localizing with LysoTracker Red, At least 100 bacteria were counted. Representative images are shown in panel b (si NT) and (si Rab35). *** represents *p*<0.001, Values shown represent mean ± standard deviation of results of three independent experiments. The graphs (panel c) show the quantification of the fluorescence intensity along the white line shown in the merged image. UPEC from si NT sample did not co-localize with LysoTracker Red; while UPEC from si Rab35 sample co-localized with LysoTracker Red. **C.** BEC cells were transfected with si NT or si Rab35. 24 h later the cells were transfected with RFP-Cathepsin D expressing plasmid. After another 24 h the cells were infected with GFP-UPEC. The colocalization between UPEC containing vacuole (UCV) and Cathepsin D was observed 24 h post infection by confocal microscopy. Results are represented as % of cathepsin D positive UCV (% of UCV that is positive for cathepsin D). At least 100 bacteria were counted. ** represents *p* < .01, Values shown represent mean ± standard deviation of results of three independent experiments. **D.** Cathepsin D protein expression was analyzed in si NT/ si Rab35 cells left uninfected or infected with UPEC for 4, 24 and 48 h by Western blotting. The experiment was repeated three times with similar results and a representative blot is shown (top panel). Quantitation of the Western blots using the software ImageJ is shown (bottom panel). Relative densitometric data (mature cathepsin D/GAPDH) is shown. Values shown represent means ± standard deviations of results of three experiments. (* p<0.05 vs siNT values at the corresponding time points).

Because UPEC intracellular survival was compromised within Rab35 knocked down cells, we examined the fate of internalized bacteria in Rab35-deficient cells. Previous reports [11] and our current data show that UPEC in QIRs reside in a LAMP1 positive compartment in bladder cells *in vivo*. We also observed that UCVs *in vitro* were positive for both LAMP1 and Rab35. The percentage of Rab35 and LAMP1 positive UCV increased from 4–24 h, but thereafter this value remained fairly constant (Fig 3A). The Mander’s overlap coefficient of Rab35 and LAMP1 on UCV was 0.65 ± 0.09 (n ≥ 50) at 24 h. Since UPEC was apparently unable to sustain its intracellular growth in the absence of Rab35, we hypothesized that the bacteria might be directed to the lysosomal compartment for degradation. To test this, we first studied the colocalization of UPEC with lysosomes in normal and Rab35-depleted cells, using lyso-Tracker Red, which stains late endosomes and lysosomes [53]. Very few UPEC colocalized with lyso-Tracker Red stained compartments in Rab35 sufficient cells. However, we observed that in Rab35-deficient cells, a significantly higher number of intracellular UPEC colocalized with lyso-Tracker Red at 24 h post infection (Fig 3B). Since LysoTracker Red cannot reliably distinguish endosomes and lysosomes, we performed further experiments to probe the exact nature of the compartment occupied by UPEC in the absence of Rab35. The lysosomal protease cathepsin D is first synthesized as an inactive precursor (procathepsin D) that is then cleaved to form active cathepsin D in lysosomes [54,55]. Hence, colocalization of UPEC with cathepsin D, and the processing status of cathepsin D, could be used as further indicators of UCV’s fusion with lysosomes. Subsequent colocalization experiments revealed a significant increase in the colocalization of UPEC with cathepsin D in Rab35-silenced cells, compared to controls (2.6 fold, *p* = 0.001; Fig 3C). In addition, we also found that the levels of mature/activated form of cathepsin D increased in Rab35 knockdown cells with the progression of infection (Fig 3D). These results indicate that UPEC are transported to the degradative lysosomal compartment in the absence of Rab35.

### Iron acquisition via the Rab35-Transferrin receptor 1 pathway is critical for the intracellular survival of UPEC within BEC

**Fig 4:**
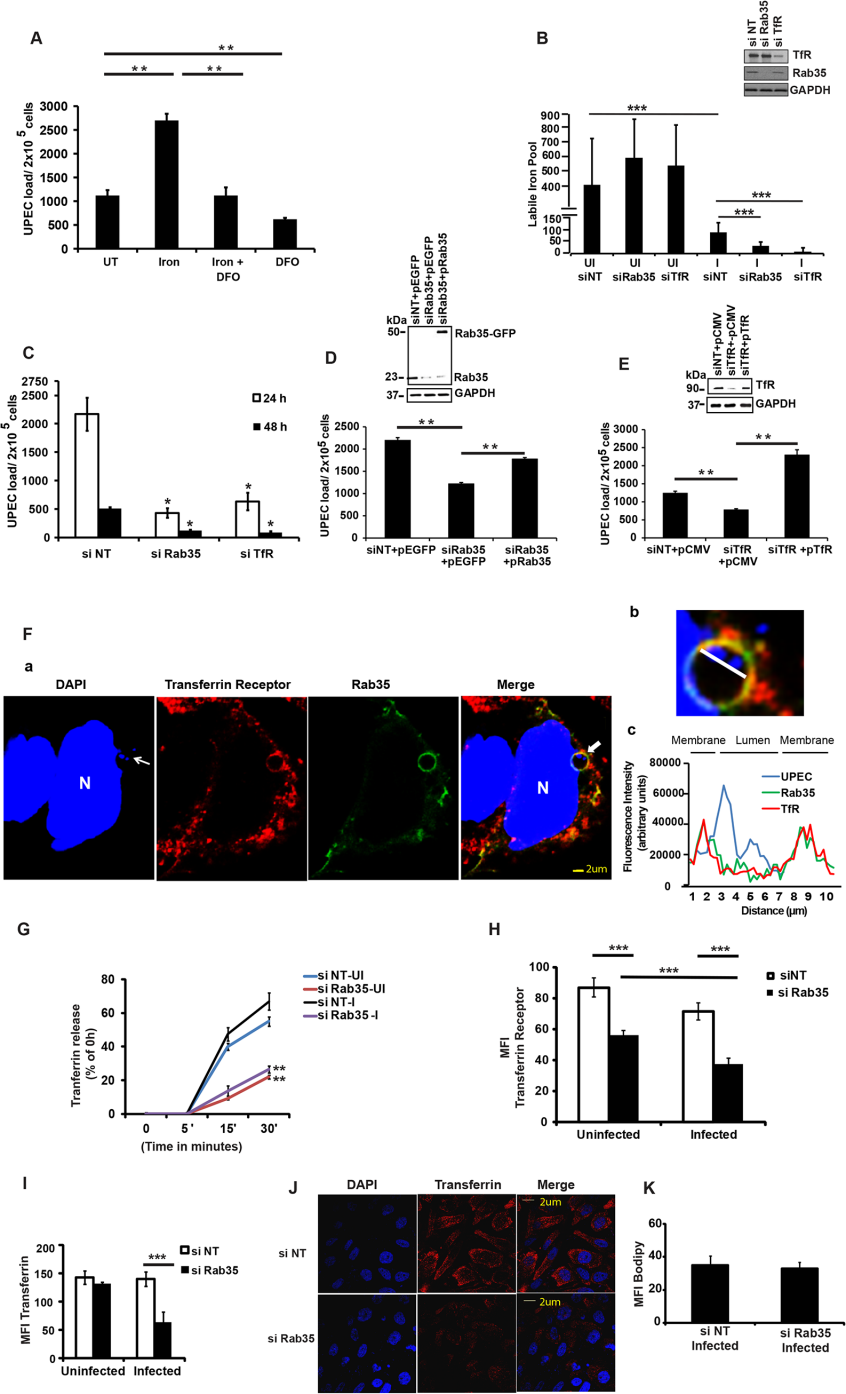
Iron acquisition *via* Rab35/transferrin receptor 1 pathway is critical for the intracellular survival of UPEC within BEC. **(A)** UPEC-Infected BEC cells were either left untreated (UT) or treated with holotransferrin (iron), deferoxamine (DFO) or iron+DFO in serum-free conditions. The intracellular bacterial load was determined 24 h post-infection by lysing the cells in 0.1% Triton X-100 and plating on LB-agar. **B** BEC cells transfected with si NT, si Rab35 or si TfR were left either uninfected or infected with UPEC. Intracellular iron levels at 24 h post infection were determined by calcein-AM fluorescence as described in materials and methods. Immunoblotting was done to confirm the knock down efficiency of Rab35 and TfR siRNA (inset panel). **C.** BEC cells were transfected with si TfR, si Rab35 or siNT for 48 h followed by infection with UPEC. Intracellular bacterial load at different time points was determined as described before. **D and E.** BEC-5637 cells were transfected with 100nM each of 3’ UTR si Rab35 (**D**) 3’ UTR si TfR (**E**). 24 h following knockdown, the cells were transfected with either empty vector control or plasmids encoding Rab35 (D) or TfR (E) for 24 h. Cells were subsequently serum starved for 4 h and infected with UPEC at MOI 500. The infected cells were finally left in Gentamycin (10 μg/ml)—RPMI with 3% serum. Intracellular bacterial load was determined at 24 h. Immunoblotting was done to confirm the knockdown and over expression efficiency (inset panels). **F.** BEC cells were transfected with GFP-Rab35 for 24 h followed by UPEC infection for 24 h; fixed, permeabilised, stained for intracellular Transferrin Receptor and analyzed by confocal microscopy. Rab35 (green), DAPI {blue, host nuclei or bacteria (UPEC)}, and Transferrin receptor (red). Arrows in the DAPI panel indicate UPEC colonies. Panel b shows the magnified image of the UCV marked by arrows in the merged image {(optical magnification (63x) and electronic zoom (3.7x)}. The graph (panel c) shows the quantification of the fluorescence intensity along the white line shown in panel b. N, host nucleus. Both Rab35 and TfR colocalized at the UCV. The experiment was repeated three times with similar results and a representative image is shown. **G.** si NT and si Rab35 cells were either left uninfected or infected with UPEC for 24 h. Subsequently, the cells were loaded with Alexa 568 labelled transferrin for 20’ at 37°C. Unbound Transferrin was removed and the cells were incubated with RPMI containing unlabelled holotransferrin for the given time points. The cells were fixed and analyzed for intracellular Transferrin by confocal microscopy. Transferrin receptor recycling (Transferrin release) was measured as % of intracellular Transferrin at 0h - intracellular Transferrin at the given time point. **H** si NT and si Rab35 cells were either left uninfected or infected with UPEC. 24 h later the cells were fixed and stained for cell surface Transferrin Receptor and represented as Mean fluorescence intensity (MFI) and analyzed by confocal microscopy. **I and J.** si NT and si Rab35 BEC cells were either left uninfected or infected with UPEC. 24 h later the cells were loaded with Alexa 568 labelled transferrin for 20’ at 37°C. After removing the unbound Transferrin the cells were fixed and analyzed for intracellular Transferrin by confocal microscopy. Mean fluorescence intensity (MFI) is shown in the graph (**I**). The confocal microscopy experiments were repeated three times with similar results and a representative Fig for the infected samples are shown in (**J**). Transferrin (red). **K.** Infected si NT and si Rab35 cells were loaded with Bodipy 493/503 at 24 h post infection. The cells were subsequently fixed and analyzed for lipid staining by confocal microscopy and represented as MFI. Data represent mean ± standard deviation of results of three independent experiments (A, B, C, D, E, G, H, I and K). * represents p<0.05, **represents p<0.01. *** represent *p*<0.001. Results of (A), (C), (D) and (E) are expressed as bacterial load/ 2x10^5^ cells. si = siRNA, si NT = negative control siRNA.

We next sought to determine the mechanism by which Rab35 is utilized by UPEC to avoid fusion of UCV with lysosomes, thus promoting its intracellular survival within BEC. Rab35 is involved in the fast recycling of TfR, the protein critical for the cellular uptake of iron, and UPEC is known to up-regulate TfR1 levels during intracellular infection of BEC [32]. Because of its involvement in TfR recycling, we hypothesized that Rab35 might be recruited by UPEC in order to acquire iron (a highly limiting nutrient) in addition to its role in avoidance of fusion with lysosomes. We performed several experiments to address this possibility. First, we re-visited the requirement of iron for the multiplication of UPEC in cell-free cultures [56]. As seen in S3A Fig, addition of iron significantly increased UPEC levels in cell-free cultures, while the addition of the iron chelating compound deferoxamine reversed this effect. Although previous studies have reported the induction of iron acquisition-associated genes in UPEC during IBC formation [32,57], the role of iron in the intracellular survival of UPEC has not yet been studied in detail. We therefore determined whether iron is critical for the intracellular survival of UPEC. We found that supplementation of iron in the form of holotransferrin indeed significantly increased the intracellular levels of UPEC in BEC cells at 24 h (2.4 fold, *p* = 0.006, Fig 4A). Conversely, treatment with deferoxamine reduced the intracellular survival of UPEC (2.4 fold, *p* = 0.004, Fig 4A). Accordingly, we also observed increased fusion of UCV with lysosomes in deferoxamine treated cells (S3B Fig).

To determine whether a Rab35-mediated pathway is exploited by UPEC to meet their iron requirements through TfR, we first analyzed the role of Rab35 and TfR1 in modulating the levels of metabolically available iron or the labile iron pool (LIP) in BEC, within the context of UPEC infection. Knock down of either Rab35 or TfR1 did not significantly alter LIP in uninfected cells at both 4 h (S4A Fig) and 24 h (Fig 4B). Notably, UPEC infection led to a severe reduction of the LIP in negative control siRNA treated BEC cells (3.26 fold, *p* = 0.001) at 24 h (Fig 4B), but not 4 h (S4A Fig), post infection. This data argues that UPEC utilize the bulk of the pre-existing LIP of BEC during infection. Remarkably, this reduction of LIP was further aggravated in both Rab35 (2.8 fold reduction in comparison to infected negative control siRNA treated samples, *p* = 0.0001) and TfR1 knocked down cells (11.4 fold reduction in comparison to infected negative control siRNA treated samples, *p* = 0.0001) at 24 h post UPEC infection (Fig 4B). Interestingly, silencing of TfR1, but not Rab35, also reduced the LIP at 4 h post-infection (S4A Fig), although UPEC load was unaffected at this time point (S4B Fig). Since the reduction of LIP upon UPEC infection was much greater in Rab35 and TfR1 silenced cells at 24 h than in infected control cells, we reasoned that UPEC is dependent on Rab35 and TfR1 to actively acquire iron from the extracellular milieu to replenish intracellular iron levels for its survival at later stages (24 h) of infection.

Further highlighting the association between Rab35 and TfR1 during UPEC infection, we found that similar to Rab35 knock down cells, the reduced iron pool in TfR1 knockdown cells (Fig 4B) was also associated with reduced survival of UPEC at 24 and 48 h post infection (3.4 fold at 24 h, *p* = 0.02, Fig 4C). These experiments demonstrate that UPEC exploits Rab35 and TfR1 to acquire iron during later stages of intracellular infection (≥ 24 h) in bladder cells.

To ensure the specificity of the siRNAs used, we also performed siRNA phenotype rescue experiments. Rab35 or TfR was knocked down using siRNAs targeting their 3’UTR regions, followed by ectopic overexpression of Rab35/TfR respective coding regions, and followed by UPEC infection. It was found that ectopic overexpression of non-degradable Rab35 (Fig 4D) or TfR (Fig 4E) rescued the growth defect observed in Rab35/TfR-deficient cells.

In order to determine the specificity of the requirement of Rab35 in meeting the iron requirement of persistent UPEC infection, we examined whether Rab14, which is also known to be involved in TfR recycling [58] is utilized by UPEC for intracellular survival. Rab14 was knocked down in BEC cells and the intracellular UPEC levels were determined at various time points (4, 24 and 48 h). As seen in S5 Fig, we found that Rab14 was not essential for the UPEC survival within BEC.

### Rab35 and Transferrin Receptor 1 co-localize at the UPEC-containing vacuole

Since both Rab35 and TfR1 are critical for UPEC intracellular survival, we hypothesized that UPEC recruits Rab35 to meet its iron nutrient requirements through the regulation of TfR transport towards bacteria containing vacuoles. To test this, we first examined whether TfR1 and Rab35 protein colocalized with UCV during infection. Confocal microscopy analysis showed that TfR1 colocalized with Rab35 at the cell surface during UPEC infection (S4C Fig). More interestingly, TfR1 shows very obvious colocalization with UPEC-containing Rab35 positive UCVs (Fig 4F) with Mander’s overlap coefficient of 0.85 ± 0.12 (n ≥ 50). To further characterize the nature of UCV, we performed colocalization studies with another marker of early endosomes (early endosomal antigen, EEA1) and found no obvious colocalization of UCV with EEA1 (S4D Fig). A recent study has reported that UPEC in QIRs *in vivo* survive within an autophagosome-like compartment [29]. Consistent with this, we found that Rab35-positive UCV *in vitro* were also associated with the autophagy marker LC3 (S6 Fig). The Mander’s overlap coefficient of Rab35 and LC3 in the infected cells was determined to be 0.60 ± 0.10 (n ≥ 50).

### Rab35 is required for the recycling and maintenance of cellular surface levels of TfR during UPEC infection

We next examined whether UPEC infection affects TfR recycling in the presence and absence of Rab35 expression. It was found that UPEC infection *per se* did not affect TfR recycling (as determined by measuring transferrin release) in Rab35 sufficient (siNT) cells compared to uninfected cells (Fig 4G). We then examined whether absence of Rab35 expression affects TfR recycling in the context of UPEC infection. Consistent with previous reports, we found that Rab35 silencing significantly reduced TfR recycling in uninfected BEC cells (2.5 fold at 30’, *p* = 0.002), compared to uninfected control siRNA treated cells (Fig 4G). Similarly, the recycling of TfR to the cell surface was significantly reduced (2.53 fold at 30', *p* = 0.002) in UPEC infected Rab35 knockdown BEC cells (Fig 4G). We also quantified the surface expression of TfR in similar conditions, using immuno-staining based fluorescence microscopy. Rab35 knockdown resulted in a 1.6-fold (*p* = 0.0001) and 1.9-fold (*p* = 0.0001) reduction in the levels of cell surface TfR in uninfected and infected cells, respectively, compared to control siRNA treated cells (Fig 4H). Although there was no significant difference in the surface expression of TfR during infection in the siNT group, there was a moderate but significant reduction (1.5-fold, *p* = 0.0007, Fig 4H) in the TfR surface expression in infected cells compared to uninfected cells in the Rab35 knockdown group, indicating a requirement for Rab35 in maintaining the TfR surface levels during UPEC infection.

Additionally, consistent with the reduction of the surface TfR receptor levels and their reduced ability to accumulate a labile iron pool within persistently infected cells, Rab35 knock-down cells also showed a significantly reduced uptake of Alexa Fluor labeled Transferrin at 24 h post infection (2.2-fold, *p* = 0.0001, Fig 4I and 4J). There was no reduction in the uptake of Alexa labeled Transferrin upon infection of wild type cells, in comparison with uninfected cells. Similarly, Rab35 knockdown alone did not reduce Transferrin uptake in uninfected cells. In order to confirm that the reduced uptake of transferrin was not due to a general defect in endocytosis due to infection, we analyzed the uptake of a lipid (MFI Bodipy) in Rab35 silenced cells. As shown in Fig 4K, UPEC infected Rab35 knockdown cells were not defective in bulk endocytosis. The above-described observations demonstrate that Rab35 is critical for the maintenance of the cellular surface levels of TfR and the subsequent iron-transferrin uptake by BEC cells during UPEC infection.

### Iron supplementation is unable to support intracellular UPEC survival in the absence of host Rab35 or TfR

**Fig 5:**
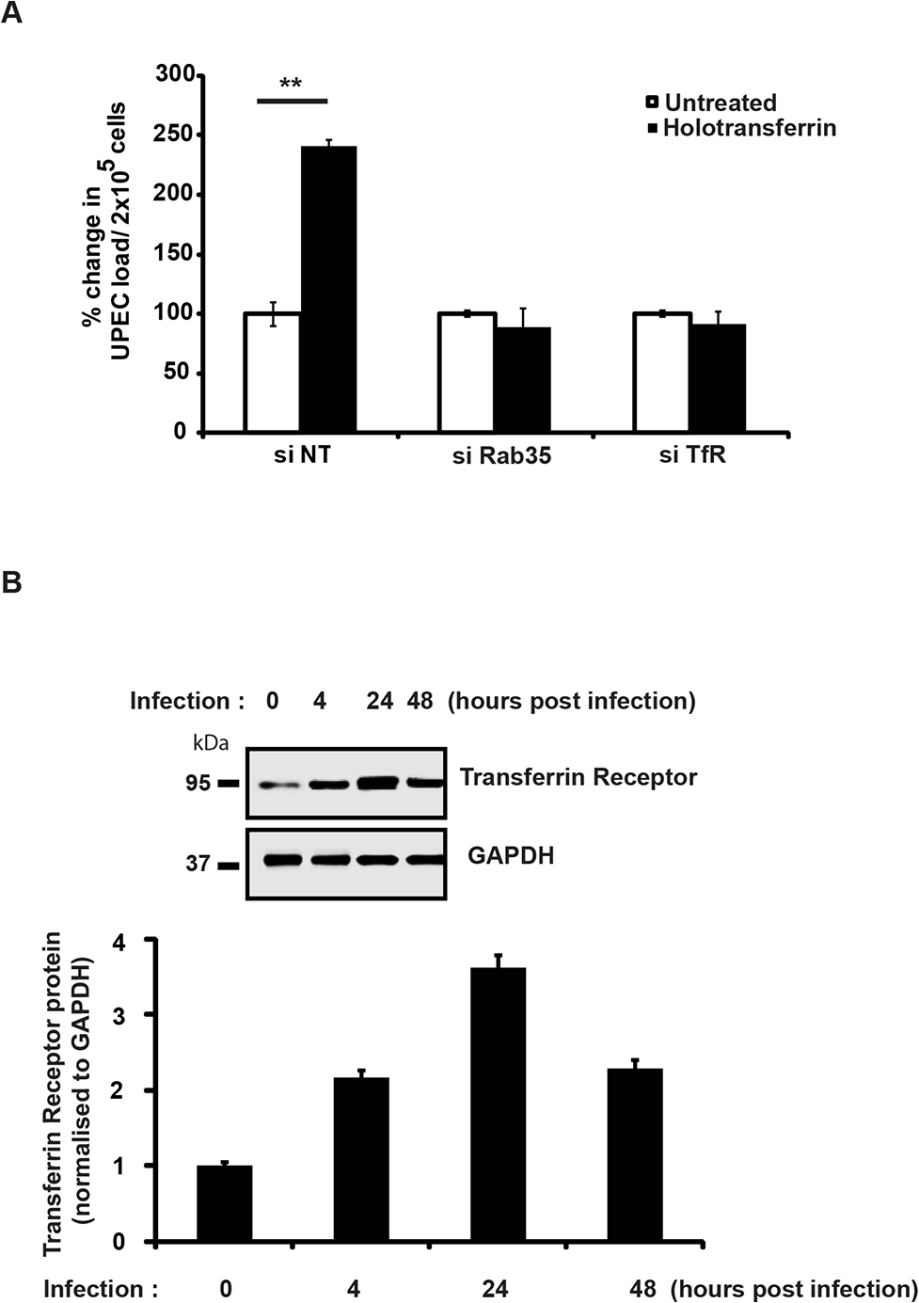
Iron supplementation is unable to support intracellular UPEC survival in the absence of Rab35 or TfR. **A. si**NT, siRab35 or siTfR treated BEC cells were maintained in serum-free conditions 4 h prior to infection and were subsequently left in serum free medium for the rest of the duration of the experiment. Cells were infected with UPEC at MOI 500 for 2 h. Following infection, and gentamycin treatment, the cells were washed and left in RPMI with gentamycin (10μg/ml) and treated with 50μg/ml holotransferrin or left untreated for further 24 h. The cells were then lysed and plated for enumeration of intracellular bacteria by cfu assay. Results are expressed as % change in bacterial load/ 2x10^5^ cells. Values shown represent means ± standard deviations of results of three experiments. (** p<0.01 vs untreated siNT values). **B.** UPEC infection upregulates TfR at the protein level. BEC cells were infected with UPEC and TfR protein expression was analyzed at 4, 24 and 48 h post infection by Western blot analysis. Quantitation of the Western blots using the software ImageJ is shown (bottom panel). Relative densitometric data (TfR/GAPDH) is shown. Values shown represent means ± standard deviations of results of three experiments. (* p<0.05 vs 0 h time point values).

The data provided earlier showed that iron supplementation by the addition holotransferrin enhanced the intracellular UPEC levels within normal cells. We next investigated whether iron supplementation can rescue the growth defect observed in Rab35/TfR-silenced cells. Holotransferrin was added to the culture media of control cells, TfR- or Rab35-knockdown cells and UPEC infected cells. We found that iron supplementation was unable to rescue the UPEC growth defect in both TfR and Rab35-knock down cells. As shown in Fig 5A, UPEC infected control (si-NT) cells showed significantly higher bacterial load in the presence of extracellular iron (holotransferrin) treatment (2.4 fold, *p* = 0.006). However the TfR/Rab35-silenced cells did not show any increase in the bacterial load even with iron supplementation, most likely due to the failure of these cells to accumulate iron from the extracellular milieu. These results collectively suggest that iron is important for UPEC survival inside bladder cells, and the Rab35-TfR pathway plays a major role in meeting the iron requirements of intracellular UPEC.

Earlier studies have shown that the expression levels of TfR are negatively regulated by intracellular iron content [59]. Since UPEC-infected cells had reduced intracellular iron levels (LIP) at 24 h post-infection (Fig 4B), we hypothesized that TfR levels might be elevated in these cells. Consistent with our hypothesis, we observed increased expression of TfR1 upon UPEC infection, with a maximal induction observed at 24 h (Fig 5B). This suggests that during infection, the observed consumption and depletion of cellular labile iron pools by UPEC will in turn trigger increased expression levels of TfR. This upregulation of TfR expression might facilitate increased internalization of Fe-Transferrin complexes into the cell.

### Genes involved in iron acquisition are differentially expressed by UPEC in the absence of Rab35

**Fig 6:**
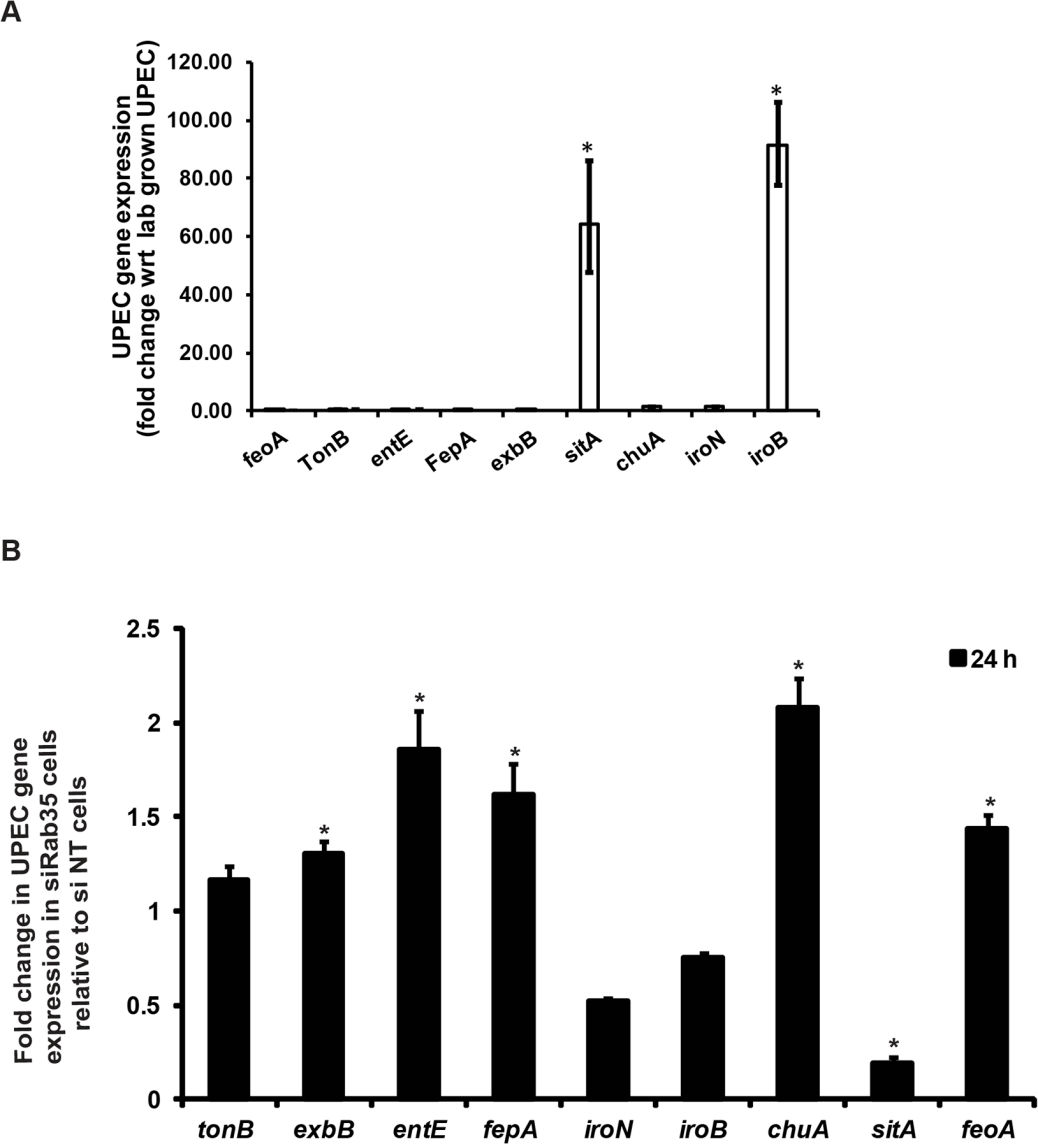
(A) and (B) Genes involved in iron acquisition are differentially expressed by UPEC in the absence of Rab35. si NT and si Rab35 cells were infected with UPEC at MOI 500. mRNA was isolated from either **(A)** cell free UPEC (represented as lab-grown UPEC) used for infecting the BEC cells or **(B)** UPEC isolated from infected cells at 24 h post infection and was analyzed for the expression of various genes involved in iron uptake by qRT-PCR. The values represent fold change in UPEC gene expression in **(A)** control (si-NT) cells relative to lab-grown UPEC or **(B)** Rab35-silenced cells relative to control (si-NT) cells * represents *p*<0.05, Values shown represent mean ± standard deviation of results of three independent experiments.

Because there is a constant struggle between the host and bacteria to secure iron for their respective uses, we wondered how the genetic regulatory system in UPEC would respond to the significant reduction in availability of cellular iron caused by Rab35 depletion. In response to iron limitation in the urinary tract, UPEC produce various siderophores (eg, EntE) and siderophore receptors (eg. FepA, IroN) as well as components of energy transduction systems required for siderophore uptake (eg. TonB, ExbB). In addition, UPEC can also utilize ferrous iron via the iron transporter FeoA and host heme stores (via ChuA, the receptor for hemin) [32,33]. We first determined the levels of several genes known to be involved in iron acquisition in UPEC (*feoA*, *tonB*, *entE*, *fepA*, *exbB*, *sitA*, *chuA*, *iroN*, and *iroB*) at 24 h post-infection in Rab35-sufficient BEC cells by qRT-PCR analysis. We found that expression of two of the nine genes tested were significantly up-regulated in UPEC at 24 h post-infection in comparison to lab grown bacteria: *sitA*, encoding an ABC type iron transporter subunit; and *iroB*, encoding a putative glycosyltransferase involved in salmochelin glucosylation (Fig 6A). In Rab35-silenced cells, five of the UPEC genes tested were up-regulated at 24 h post-infection: *exbB*, *entE*, *fepA*, *chuA*, and *feoA* (Fig 6B). Intriguingly, the expression of the two genes *sitA* and *iroB* that were up-regulated in infected control cells were found to be either down-regulated (*sitA*) or did not change significantly (*iroB*) in Rab35 silenced cells at 24 h post-infection. Thus, intracellular UPEC display a specific response to iron limitation resulting from the abrogation of Rab35 expression, further supporting a role for Rab35 in facilitating iron acquisition during intracellular UPEC infection.

## Discussion

Numerous studies have described the complexities and consequences of intracellular UPEC infections in mice, humans, and cell culture. In general, studies on intracellular UPEC infections resembling those found *in vivo* have been limited partly because *in vitro* models do not seem to recreate the same structures. Several features of *in vitro* infections of cultured 5637 cells, however, have been validated using mouse models of infection, particularly for early stages of invasion [60,61]. We have used this *in vitro* system to identify a role for host Rab35 in intracellular UPEC survival.

At late time points (> 2 weeks) during experimental mouse infections, UPEC is reported to reside within LAMP1-positive and Cathepsin D-negative compartments (resembling late endosomes) *in vivo* [11,62]. How UPEC survive within these acidic environments and how the phagosome is modified or phagosome maturation is arrested has yet to be fully determined. In our *in vitro* cell culture infection system, we also found that UCVs are LAMP1 positive. The UCVs are also positive for Rab35 and TfR; therefore, the UCV compartment shows some characteristics of late endosome and early/recycling endosomes. Accordingly, we found that in experimentally infected mice, UPEC within the uroepithelium also colocalize with Rab35 and the QIR marker LAMP1. The observation that UCV’s were also associated with the autophagy marker LC3 also indicate that UCV may also possess some characteristics of autophagosomes, although more studies are required to characterize this further. We therefore add Rab35 to the list of QIR markers *in vivo* (LAMP1-positive, LC3-positive, ATG16L1-positive, and Cathepsin D-negative) and for the first time demonstrate that UCVs found during *in vitro* infection of 5637 cells carry similar vesicular markers, suggesting that intracellular infection of 5637 cells may be useful as a model for QIR development and therefore recurrent UTI.

**Fig 7:**
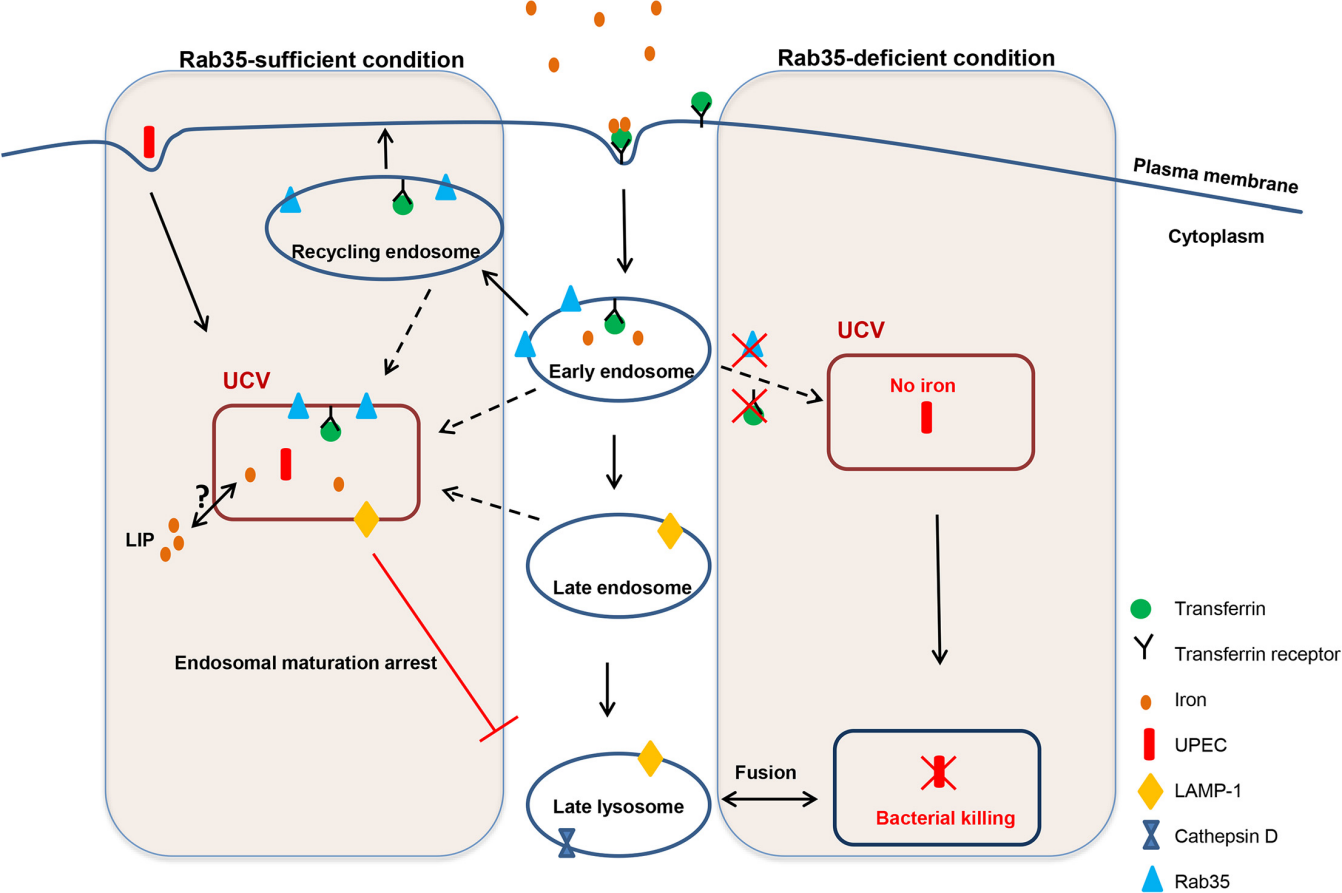
Schematic representation of how the presence or absence of Rab35 affects the fate of UPEC survival within UCV. In Rab35-sufficient conditions, UCV fuses or acquires the markers for early/recycling endosomes (Rab35, TfR) and late endosomes (LAMP1), acquires iron and prevents the fusion of UCV with late lysosomes leading to UPEC survival. In the Rab35-deficient conditions, the UCV is unable to acquire Rab35/TfR and iron and subsequently UPEC gets killed by fusion of UCV with late lysosomes.

Survival of UPEC within UCVs carrying these unique vesicular markers could be one of the mechanisms by which the bacteria avoid endosomal maturation/lysosomal fusion. It is also worth noting that Rab35 deficiency resulted in reduced survival of UPEC within bladder cells in culture and this is supported by the observation of an increased percentage of UPEC-containing Cathepsin D-positive compartments (late lysosomal/phagolysosomal) upon Rab35 silencing. The percentage of UCV positive for Rab35 continuously increased throughout the course of infection further highlighting the requirement of this pathway for UPEC persistence within bladder cells. A model for how Rab35 promotes UPEC survival within BEC is given in Fig 7. The compartment occupied by UPEC resembles that of *A*. *phagocytophilum* where the bacterium has been shown to preferentially recruit Rab GTPases (including Rab35) associated with the endocytic recycling pathway to its vacuole in order to facilitate intracellular survival [50]. For the intracellular bacteria *M*. *avium* [44] and *M*. *tuberculosis* [63], bacteria-containing phagosomes are also known to fuse with recycling/early endosomes to acquire iron and to halt the phagosome maturation process. The recruitment of host Rab35 to the UCV observed during UPEC infection hints at the possibility that it is a bacteria-induced phenomenon. Future studies will focus on the exact mechanism by which UCV hijacks Rab35 to its vacuole and the bacterial proteins (if any) that are involved in mediating this process.

As a part of host defense mechanisms against bacterial infection, mucosal surfaces like the urinary bladder mucosa often limit the availability of essential nutrients, such as iron. To counteract this, bacteria including UPEC have developed or acquired apparently redundant systems to acquire free or host protein-bound iron, including siderophores, outer membrane iron compound receptors and TonB-dependent receptors. At least one of the two major siderophores (enterobactin or aerobactin) were required for the efficient host colonization of UPEC [38]. Our study provides a preliminary understanding of the expression status of various UPEC genes associated with iron acquisition during intracellular survival of UPEC within urinary bladder cells. It is interesting to note that only two bacterial genes, *sitA* and *iroB* (out of 9 tested), were significantly up-regulated in intracellular UPEC infection in comparison to the extracellular form. Intriguingly, UPEC in Rab35 silenced cells showed a significant down-regulation in the expression of *sitA*, while the expression of *iroB* remained unchanged. The significance of this observation is currently unclear; although functional redundancy of the iron acquisition systems in UPEC [64] could be one of the contributing factors. In contrast, the Rab35 silenced cells showed an increase in the mRNA levels of genes such as *tonB*, *fepA* (a TonB-dependent active transporter that recognizes extracellular ferric enterobactin and translocates it into the periplasm), *entE* (enterobactin synthetase), *chuA* (heme-derived ferric iron receptor), *feoA* (ferrous iron transport protein), and *exbB* (component of TonB complex). Increases in the mRNA levels of these genes were also previously reported in a comparison of UPEC in IBCs with UPEC in the distal intestine, further supporting their role in iron acquisition by intracellular UPEC [32].

Until now, only a few reports have examined the requirement of bacterial iron acquisition systems in the extracellular and IBC forms of UPEC [32–35], and there has been only one study to report the up-regulation of host genes associated with iron regulation (*TfR* and *Lcn2*) in the urothelial cells associated with IBCs [32]. Our study demonstrates for the first time that UPEC adopts a unique strategy by exploiting the fast endocytic recycling pathway component Rab35 to sequester TfR, in turn will increasing the availability of iron for the intracellular UPEC. In the absence of Rab35 expression, the TfR will not be efficiently recycled to the cell surface, leading to insufficient replenishment of intracellular iron, ultimately hampering the growth of UPEC (by the fusion of UCV with lysosomal compartments). The iron supplementation experiments confirmed the notion that iron is critical for the survival of both extracellular and intracellular UPEC. The intracellular labile iron pool within bladder epithelial cells decreased to ~38% within the first 24 h following UPEC infection, suggesting that UPEC derives majority of its iron from the labile iron pool of the host cell. Two major sources for the labile iron pool are (i) transferrin-bound iron, internalized via transferrin receptors on the cell surface and (ii) ferritin, the intracellular iron storage protein. Silencing of TfR almost completely eliminated the labile iron pool at 24 h, suggesting that this is the major pathway by which the iron pool is maintained within the UPEC-infected cell. In further support of this, silencing of Rab35 also reduced intracellular iron. The fact that Rab35/TfR-silenced cells were unable to take up extracellular iron and support UPEC growth also illustrates the requirement of Rab35 in transferrin receptor-mediated iron uptake during UPEC infection. TfR level is controlled by levels of intracellular iron. When the cellular iron pool drops, iron-regulatory proteins IRP1 and IRP2 are activated, and they bind iron-responsive elements (IRE) to upregulate TfR by a post-transcriptional mRNA stability mechanism [65]. We have indeed observed an increased TfR protein expression during intracellular UPEC infection.

Since our studies have identified that intracellular UPEC utilize iron for survival within BEC, a major question that remains to be resolved is the source of iron utilized by the Rab35/TfR positive intravacuolar bacteria. Iron taken up by cells through the TfR-Tf complex first reaches endosomes from where it is exported into cytoplasm, leading to the formation of the cellular labile iron pool. Thus, intravacuolar pathogens can acquire iron from two possible sources: (a) extracellular (transferrin-bound iron) that is used “directly” within the vacuole; or (b) intracellular (the cytoplasmic labile iron pool). Future studies will determine whether Rab35/TfR+ve UCV utilizes vacuolar iron or the cytoplasmic labile iron pool.

Given the molecular similarities with QIRs, UCVs may also contain UPEC that are not actively replicating. If the acquired iron is not utilized for growth, an interesting question that remains to be resolved is the purpose of acquisition of iron by UPEC at this stage. UPEC at this stage could be comparable to intravacuolar persistent stage of *M*. *tuberculosis* where the bacterium attains a seemingly non-replicating (quiescent) or slowly replicating state of growth [66]. Bacteria in a quiescent state display nominal metabolic capacity, maintain membrane potential, and do not undergo obvious morphological differentiation [67,68]. This state allows the bacteria to escape from host-stress responses such as low iron, low oxygen, and low pH, and to maintain a viable bacterial population during the stress period. When conditions are favorable, these bacteria will then resume growth and start multiplying actively. Our studies show that iron is one of the major limiting nutrients that UPEC exploits to survive within the vacuole of BEC. The iron sequestered by the UPEC may be used for maintaining the basal metabolism of the persistent bacteria. In support of this notion, our data shows that iron depletion reduces the survival of UPEC, while supplementation of iron triggers multiplication/ bacterial growth.

On the other hand, it should be noted that too much iron is also detrimental to the bacteria, since iron levels and oxidative stress are directly linked [69]. Iron itself is a mediator of oxidative stress, as it chemically generates hydroxyl radicals that are capable of damaging DNA and proteins [70,71]. To prevent iron-dependent cytotoxicity, bacteria also store excess iron (similar to the host) with ferritin, bacterioferritin, and Dps proteins [72] to be utilized when conditions are favorable. Thus the bacterial iron storage proteins store iron as well as protect the bacteria from iron-mediated oxidative stress conditions. Supporting this, it was reported that ferritins, Dps, or bacterioferritins enhance the growth of iron-deprived pathogens such as *E*. *coli* [73], *Campylobacter jejuni* [74], and *Mycobacterium tuberculosis* [75], protect them against oxidative stress and support their survival within the host [76]. UPEC in the UCVs could therefore be actively sequestering iron from the host to meet one or more of the following purposes: (1) maintaining its basal metabolism and survival during stressed conditions (low iron, low oxygen, or low pH); (2) sequestering excessive Fe as a stress resistance mechanism as well as for storage; and (3) for active multiplication when conditions are favorable. Further studies are necessary to distinguish among these possibilities. There is therefore a delicate balance between the host response (by sequestering iron to prevent bacterial multiplication) and the bacterial response (by exploiting iron to a level at which it is not toxic to the bacteria).

The identification and characterization of Rab35 as a critical regulator of UPEC intracellular survival may open up new avenues for the therapeutic intervention for the elimination of chronic or persistent UPEC infections.

## Materials and Methods

### Ethics statement

All animal experiments were performed in accordance with protocols that were reviewed and approved by the A*STAR Biological Resource Center Institutional Animal Care and Use Committee (protocol #130853). These protocols were approved to be in accordance with the prevailing Singapore National Advisory Committee for Laboratory Animal Research (NACLAR) guidelines.

### Bacterial strain and growth conditions

The clinical UTI isolate E. coli CI5 strain [77,78] was kindly provided by Prof. Soman N Abraham (Duke and Duke-NUS) and was used throughout the *in vitro* study. The prototypic cystitis strain UTI89 [52] transformed with plasmid pANT4 expressing GFP [79] (referred to as strain SLC-295) was used for *in vivo* infection experiments in mice. The K12/mCherry strain (SLC-720) is E. coli K12 substr. MG1655 transformed with an mCherry expression vector (pSLC-229). pSLC-229 was generated by replacing the GFP gene in plasmid pANT4 [79] with mCherry amplified from pXJ40_2 (a kind gift from Sohail Ahmed). Briefly, mCherry was amplified using PCR (using the primers 5’-GACTCTGAATTCATGGTGAGCAAGGGCGAGGA and 5’-GATCCTGCATGCTTACTTGTACAGCTCGTCCATGC). The expected 700bp product was digested with EcoRI and SphI and cloned into the same sites of pANT4 to yield pSLC-229; the correct predicted sequence was verified by sequencing the ligation junctions and the entire mCherry gene. pSLC-229 was then transformed into MG1655 to yield SLC-720.

### Cell line and growth conditions

Human Bladder epithelial cell line 5637 (ATCC HTB-9) was maintained at 37°C and 5% CO2 in RPMI 1640 media (Gibco) supplemented with 10% Fetal Calf Serum (Gibco).

### Plasmid constructs

All the Rab GTPase plasmids used in this study including the EGFP tagged wild type Rab35, and mutants (Rab35 Q67l and Rab35 S22N) were as described before [50]. The other GFP/RFP tagged Rab GTPases used for the microscopy-based study were: Rab1, Rab2A, Rab3A, Rab4A, Rab5, Rab6A, Rab8A, Rab10, Rab11A, Rab14, Rab18, Rab22A, Rab27A, and Rab33A. RFP-tagged LAMP1 and Cathepsin D plasmids were obtained from (Addgene). Transferrin receptor plasmid was a kind gift from Dr. Kuni Matsumoto.

### Antibodies

Human Rab35 (9690) and Cathepsin D antibody (2284) were obtained from Cell Signaling Technology. Transferrin Receptor (sc-32271)) and LAMP1 (sc-20011) antibodies was obtained from Santa Cruz. Anti GFP antibody (ab540) was obtained from Abcam. Mouse Rab35 (NB20042) antibody was purchased from Novus Biologicals. Rabbit polyclonal antibody to LC3 (ab58610) was purchased from Abcam. Mouse LAMP1 antibody was obtained from Abcam (ab25245).

### Intracellular bacterial survival assay

BEC 5637 cell monolayers grown in 24-well tissue culture plates were infected with CI5 UPEC at a multiplicity of infection of 500 bacteria per host cell. For generation of heat-killed UPEC, the bacteria were suspended in PBS and heated at 100°C for 30 min. The non-viability of bacteria was confirmed by plating the bacteria on LB agar plates. To facilitate and synchronize bacterial contact with the host cells, plates were centrifuged at 600 × g for 5 min. After 2 h incubation at 37°C, cells were washed three times with RPMI to remove nonadherent bacteria. Monolayers were then incubated for 1 h with complete RPMI medium plus 100 μg/ml of gentamicin (Gibco) to kill extracellular bacteria. Subsequently cells were washed and further incubated in fresh medium containing gentamicin (10 μg/ml) for the entire duration of the experiment. At designated times monolayers were washed with PBS and subsequently lysed in PBS plus 0.1% Triton X-100, and bacteria present within the lysates were enumerated by plating serial dilutions on LB agar plates. To check for invasion, cells were washed with PBS directly after 1h of 100 μg/ml of gentamicin treatment and lysed in PBS-Triton–X 100.

### UPEC exocytosis assay

Bacteria exocytosis assay was performed as described before [19]. Briefly, cells were infected as described above. After Gentamycin (100μg/ml) treatment, cells were washed twice and left in fresh culture medium containing 100mM methyl-α-D-mannopyranoside (to prevent reattachment and entry of bacteria into BEC). At indicated time points, the culture medium was collected and plated for CFU counts.

### siRNA, overexpression and transfection studies

The small interfering RNA targeting human Rab35 (5’-GCUCACGAAGAACAGUAAA-3’) [80] was purchased from Sigma. Two siRNAs targeting human Transferrin Receptor (5’-GCACAGCUCUCCUAUUGAA-3’ and 5’-GCUGAAAGCUUAAAUGCAA-3’ [81] were purchased from SABio. The TfR siRNAs were pooled and used for experiments. The siRNAs targeting the 3’UTR of Rab35 were (5’-GCGAGGGUGUGCUUGCAAAUU-3’ and 5’-GCAAAUUCAAGCAAUAAGAUU-3’) and siRNAs targeting the 3’UTR of TfR were (5’-CAGAAACCAGTTATGTGAATGATCT-3’, 5’- GGTTCAACTGTTGATTGCAGGAATA-3’, 5’-CAGACTCAGTTTGTCAGACTTTAAA-3’, and 5’- TCGGAGACAGTGATCTCCATATGTT-3’). The siRNAs were pooled and used for the experiments. The non-targeting siRNA (si-NT) from SABio was used as a negative control. Cells were transfected with siRNA using Lipofectamine 2000 (Invitrogen) according to manufacturer’s instructions and were used for further experiments 48 h post transfection.

For transfection experiments, BEC-5637 cells were plated in a 24 well plate at a density of 1 x 10^5^ cells/ well. Next day cells were transfected with 1 μg DNA using Polyethylenimine (PEI) (1:3 ratio DNA: PEI). Cells were washed 6 h after transfection and incubated in fresh culture medium for 20 h, before proceeding for further experiments.

For siRNA rescue experiments, BEC cells were transfected with 3’ UTR siRNA against Rab35 or TfR using Lipofectamine 3000 (Invitrogen) according to manufacturer’s instructions. 24 h later cells were washed and transfected with 1μg control plasmid (pEGFPC1 for Rab35 plasmid and pCMV-Flag for TfR plasmid) or Rab35/TfR expression plasmids. 24 h later cells were washed and left in serum free media for 4 h. Cells were subsequently infected with UPEC (MOI 500) under serum free conditions. The cells were finally left in 10 μg/ml Gentamycin—RPMI with 3% serum. 24 h later the intracellular bacterial load was determined as described earlier.

### Confocal microscopy

Bacterial colocalization with various markers was performed using Confocal Microscopy. Briefly, cells were grown on coverslips and transfected with plasmids for various fluorescently tagged proteins as described above. After 20 h cells were washed and infected with UPEC. At different time points, cells were fixed with 4% paraformaldehyde and stained for nuclei with DAPI. The coverslips were mounted using Prolong Gold Antifade (Molecular Probes) and examined using Zeiss confocal Microscope with appropriate filter sets. For cell surface colocalization of Rab35 and Transferrin receptor, cells were transfected with GFP Rab35 expressing plasmid. After infection the cells were fixed and cell surface Transferrin Receptor was stained with transferrin receptor antibody. The samples were analyzed by confocal microscopy as mentioned above. For the colocalization of bacteria with lysosomal compartment, infected cells were treated with LysoTracker Red (Life Technologies) for 2 h at 37°C. Cells were then fixed and analyzed by confocal microscopy. All the images were acquired on LSM 710 Carl Zeiss microscope using Plan-Apochromat 63x/1.40 oil DIC objective. Images were acquired at 16 bit depth at a resolution of 1024x1024 pixels. Z sections of images were acquired at 63X magnification and were analyzed using Zen 2010 software and were processed using Adobe photoshop7 software. For images with XZ/ YZ projections, serial Z sections of the Z stack were taken at 0.5μm thickness.

### Quantitative colocalization analysis

The selected UCVs, containing detectable Rab35 and membrane marker were used for pixel quantification and statistical analysis. Quantification of the colocalization of Rab35 with LAMP1 or TfR was done using Zen 2010 software (Zeiss), which calculates the Mander’s overlap coefficient (*R*) [82]. The values for the overlap coefficient range from 0 to 1. An Overlap Coefficient with a value of 1 indicates high colocalization and 0 represents low colocalization. The overlap coefficients were calculated using data from at least 3 independent experiments (n ≥ 50).

### Transferrin uptake and transferrin receptor recycling

Cells grown on coverslips were analyzed for transferrin uptake at 24 h post infection. Cells were washed with RPMI and were incubated in serum free media for 1h to remove any traces of Transferrin and then were exposed to 50 μg/ml tansferrin conjugated to Alexa Flour 548 (invitrogen) at 37°C for 20’. Internalization was stopped by chilling the cells on ice. External transferrin was removed by washing with PBS, which was followed with washes with PBS (pH 5) to remove bound transferrin. Cells were finally washed with PBS (pH 7) and analyzed for transferrin uptake by confocal microscopy as described above. Fluorescence intensity was analyzed by Zen software.

For Transferrin recycling cells were first allowed to uptake Transferrin as mentioned above. Following final wash with PBS (pH 7), cells were incubated in RPMI in presence of 50μg/ml holotransferrin. Cells were fixed with 4% paraformaldehyde after 5, 15 and 30 minutes. Intracellular Transferrin was analyzed by confocal microscopy as described above. Transferrin receptor recycling/ Transferrin release was measured as (Intracellular Transferrin at 0h - Intracellular Transferrin at that time point).

### RNA isolation from host cells and bacteria

At designated time points, BEC-5637 cells were lysed in RLT buffer (Qiagen) and RNA was isolated using RNeasy kit (Qiagen). For isolating RNA from bacteria from infected BEC-5637, cells were seeded and infected in 6 well plates in triplicates. At designated time points, cells were lysed by passage through 20G needle 6–7 times. The lysate was spun at 1000xg for 5 minutes to remove the cell debris. The supernatant was then spun at 10,000xg for 20 minutes to pellet down the bacteria. The bacterial pellet was subsequently lysed in RLT buffer and processed for RNA isolation as described above. cDNA synthesis was carried out by iScript cDNA synthesis kit (Bio-Rad) as per manufacturer’s instructions.

### PCR amplification of Rab35 and bacterial genes

Rab35 and the bacterial genes were amplified from cDNA by q PCR. Primer sequences for iron-related genes of UPEC were as described before [32]. Sequences for all other primers used are listed in the S1 Table.

Reactions were performed in Roche light cycler 480 Real-time PCR system under following conditions—94°C/2 minutes for 1 cycle; 94°C/30 seconds, 55°C/30seconds, 72°C/60 seconds for 40 cycles; 72°C/7 minutes for 1 cycle. Results were analyzed using delta-delta-Ct algorithm.

### Protein expression analysis

For protein expression analysis cells were lysed at 4°C for 15 minutes in RIPA buffer—(20 mM Tris-HCl (pH 7.5),150 mM NaCl, 1 mM Na2EDTA, 1% NP-40, 1% sodium deoxycholate, 2.5 mM sodium pyrophosphate) containing protease and phosphatase inhibitor cocktail (Roche). The supernatant of the lysate spun at 12000 g for 10’ was estimated for protein concentration using Lowry assay. Equal protein concentrations were run on 12% SDS gel and analyzed for protein expression by Western blotting.

### Iron uptake assay

Labile iron pool was calculated by using standard Calcein-AM protocol [83]. Briefly cells were washed with PBS-BSA (1mg/ml)-Hepes (20mM) and then treated with 0.15uM Calcein (life technologies) in PBS-BSA-Hepes for 10’ at 37°C. Cells were washed thrice with PBS-BSA (1mg/ml)-Hepes (20mM) and resuspended in same buffer. Fluorescence was measured in iTecan Fluorimeter at Excitation- 488nm, Emission- 520nm for 10 minutes to establish stable baseline signal (F1). After this, 1000uM Dipyridyl (Sigma) in PBS-BSA (1mg/ml)-Hepes (20mM) was added to cells. Fluorescence was measured again until a stable base line signal was established (F2). Labile Iron Pool was calculated as F2-F1.

### Holotransferrin/deferroxamine treatment of BEC-5637

siNT, siRab35 and siTfR cells were infected with UPEC under serum free conditions. After gentamycin (100μg/ml) treatment, cells were left in 10μg/ml gentamycin containing RPMI alone or along with Holotransferrin (50μg/ml) or deferroxamine (100μM). The bacterial load was enumerated at 24 h post infection.

### Iron treatment of UPEC

UPEC cultures were diluted to OD600 of 0.01 in 3 ml RPMI medium with vehicle control, 10μM Ferric chloride (Sigma) or 200μM deferoxamine. Bacteria were grown at 37°C with shaking. OD600 was recorded at serial intervals.

### *In vivo* mouse experiments

A GFP-expressing derivative of UTI89 (SLC-295) was grown at 37°C in 10mL of LB media without shaking for 24h, followed by a 1:1000 dilution and a second 24h of growth at 37°C in LB without shaking. Bacteria were harvested by centrifugation and resuspended in PBS to an OD600 of 0.5 (1–2×10^7^ CFU/50μL) and used directly as the inoculum. 7–8 week old female C57BL/6 mice (In Vivos, Singapore) were transurethrally inoculated with 50μL of this inoculum or sterile PBS (negative control). At 6h (5 mice), 24 h (5 mice) or 2 weeks (5 mice) post-infection, mice were sacrificed and their bladders aseptically harvested. Bladders were then hemisected and incubated in 10% formalin at 4°C overnight.

### Histologic analysis and immunofluorescence assay

The processing of the bladder samples was carried out at the A*STAR/IMCB histology facility (A*STAR, Singapore). Briefly, the bladder tissues were embedded in 2% agar for paraffin processing. For IFA, 4- to 5μm serial sections were cut longitudinally, deparaffinized in xylene (twice for 5 min at room temperature), rehydrated in 100% ethanol (twice for 2 min at room temperature), 95% ethanol (for 1minute), and 80% ethanol (for 1 minute). Antigen retrieval was performed by boiling slides for 30 min in 1mM EDTA buffer, pH 8. Slides were blocked in 1% FBS 0.4% TritonX-100 for 1 h at room temperature, and subsequently incubated overnight at 4°C with the following primary antibodies- goat anti GFP antibody (Abcam 1:500 dilution in blocking buffer) and rabbit anti Rab35 antibody (Novus biological, 1in 50 dilution in blocking buffer). For LAMP1 triple staining, the sections were additionally stained with rat anti LAMP1 antibody (abcam 1:200 blocking buffer). Sections were washed thrice with blocking buffer and were subsequently incubated with Alexa Fluor 488 (anti-rabbit), Alexa 594 (anti-goat) and Alexa 350 (anti-rat) conjugated secondary antibodies (1:500; Molecular Probes). The sections were then stained with DAPI and were analyzed by confocal microscopy.

### Statistics

P values were determined by unpaired two-tailed Student’s *t* test. P values less than 0.05 were considered statistically significant.

### Accession numbers

RAB35 (Species: Homo sapiens, Gene ID: 11021)

Transferrin receptor (Species: Homo sapiens, Gene ID: 7037)

## Supporting Information

S1 Fig A. Selective recruitment of Rab GTPases to UCV at 24 h post-infection. BEC cells were transfected with RFP/GFP tagged RabGTPases. At 24 h post transfection the cells were infected {with RFP/GFP-UPEC (MOI 500)} and the localization of Rab GTPases with UPEC-containing vacuole (UCV) was analyzed at 24 h post-infection by confocal microscopy. Number of RFP/GFP positive UCV was divided by the total number of UCVs and is represented in the graph as % of Rab positive UCV. B. **Colocalization of Rab35 with UPEC.** BEC cells were transfected with GFP-Rab35. At 24 h post transfection the cells were infected with RFP-UPEC (MOI 500) and the colocalization of UPEC with Rab35 (indicated by arrow in the merged panel) was analyzed at 24 h post-infection by confocal microscopy. DAPI (blue), UPEC (red), Rab35 (green). C. **GFP does not localize to UCV.** BEC cells overexpressing GFP were infected with RFP-UPEC (MOI 500) for 24 h and analyzed by confocal microscopy. DAPI (blue), GFP (green), and UPEC (red). Also shown at the right side are the orthogonal sections of intracellular bacteria in XZ and YZ plane. White lines represent regions in which XYZ sections were taken. Scale bar denotes 2μm. D. **Type1-pili expressing *E*. *coli* (K12) do not recruit Rab35**. BEC cells overexpressing Rab35-GFP were infected with mCherry-K12 (MOI 500) for 24 h and analyzed by confocal microscopy. DAPI (blue), Rab35 (green), and mCherry-K12 (red). E. **Heat-killed UPEC does not recruit Rab35**. BEC cells overexpressing Rab35-GFP were infected with heat-killed UPEC (MOI 500) for 24 h and analyzed by confocal microscopy. DAPI (blue, host nuclei or bacteria), and Rab35 (green). Arrows in DAPI panel indicate heat killed UPEC. Experiments were repeated three times with similar results. Representative images are shown. F. **UPEC infected mouse bladder sections showing intracellular UPEC that are negative for Rab35.** C57BL/6 mice were infected transurethrally with UPEC (UTI89 strain). Mouse bladders were removed at 2 weeks post infection and the tissue sections were processed for immunofluorescence. Green (Rab35) UPEC (red) and DAPI (blue). n = 4 sections/mouse bladder, n = 3 mice per experiment.
(TIF)

S2 Fig A. *In vivo* QIRs are positive for both LAMP1 and Rab35. C57BL/6 mice were infected transurethrally with UPEC (UTI89 strain). Mouse bladders were removed 24 h and 2 weeks post infection and the tissue sections were processed for immunofluorescence. Rab35 (blue), UPEC (red) and LAMP1 (green). B. **Rab35 associates with IBC forms of UPEC in mouse bladder sections**. C57BL/6 mice were infected transurethrally with UPEC (UTI89 strain). Mouse bladders were removed 6 h post infection and the tissue sections were processed for immunofluorescence. Rab35 (green), UPEC (red) and DAPI (blue). n = 4 sections/mouse bladder, n = 3 mice per experiment. C. **Rab35 silencing does not enhance the efflux rate of UPEC from BEC-5637 at 4 h post-infection.** BEC-5637 cells were transfected with 100nM each of si Rab35 or non-targeting siRNA (si NT). 48 h following knockdown, the cells were infected with UPEC at MOI 500. After gentamycin (100μg/ml) treatment, cells were washed in left in fresh culture medium containing 100mM methyl-D-mannopyranoside. At 4 h post infection, the culture medium was collected and plated for CFU counts as described in Materials and Methods. Results are expressed % exocytosis relative to siNT cells. Values shown represent mean ± standard deviation of results of three independent experiments.
(TIF)

S3 Fig Iron is required for UPEC growth in the cell-free system. A. UPEC grown in cell-free system (LB media) was supplemented with iron (ferric chloride) or iron chelator deferoxamine for various time points. OD600 was measured at the corresponding time points and plotted as a measure of the UPEC growth. ** represents *p*<0.01, Values shown represent mean ± standard deviation of results of three independent experiments. B. BEC cells were maintained in serum-free conditions 4 h prior to infection and were subsequently left in serum free medium for the rest of the duration of the experiment. Cells were infected with UPEC at MOI 500 for 2 h. Following infection, and gentamycin treatment, the cells were washed and left in RPMI with gentamycin (10μg/ml) and either left untreated or treated with 100μg/ml Deferoxamine (DFO). Twenty four h later, the cells were stained with LysoTracker Red for 2 h. Subsequently the cells were fixed and analyzed for colocalization between UPEC and LysoTracker Red by confocal microscopy. Graph (left panel) is depicted as % of bacteria co-localizing with LysoTracker Red. At least 100 bacteria were counted. ** represents *p*< 0.01, Values shown represent mean ± standard deviation. A representative image is shown. Colocalization between UPEC and Lysotracker red is indicated by arrow in the merged image. The graph (right panel) shows the quantification of the fluorescence intensity along the white line.
(TIF)

S4 Fig A. Transferrin receptor, but not Rab35 is required for the maintenance of labile iron pool (LIP) at 4 h post-UPEC infection. BEC cells transfected with si NT, si Rab35 or si TfR were left either uninfected or infected with UPEC. Intracellular iron levels at 4 h post infection were determined by calcein-AM fluorescence as described in materials and methods. Data represent mean ± standard deviation of results of three independent experiments **represents p<0.01. si = siRNA, si NT = negative control siRNA. B. UPEC load does not change in Rab35 or TfR knockdown cells at 4 h post-infection. BEC cells were transfected with si TfR, si Rab35 or siNT for 48 h followed by infection with UPEC for 4 h. Intracellular bacterial load was determined as described before. Results are expressed as bacterial load/ 2x10^5^ cells. C. Rab35 colocalizes with Transferrin receptor at the cell surface during UPEC infection. BEC cells were transfected with GFP-Rab35 for 24 h followed by UPEC infection. At 24 h post infection the cells were fixed and stained for either cell surface Transferrin Receptor. The colocalization of Rab35 with cell surface Transferrin Receptor was analyzed by confocal microscopy. D. UCV does not colocalize with EEA1. BEC cells were transfected with GFP-EEA1 for 24 h followed by infection with RFP-UPEC. At 24 h post infection the cells were fixed and analyzed by confocal microscopy. The experiment was repeated three times with similar results and a representative Fig is shown. EEA1 (green), and UPEC (red). The experiment was repeated three times with similar results and a representative Fig is shown.
(TIF)

S5 Fig Rab14 is not required for the survival of UPEC within BEC 5637. BEC5637 cells were transfected with 100nM each of si Rab14 or si NT. 48h following knockdown, the cells were infected with UPEC at MOI 500. Intracellular bacterial load at different time points {4 h (invasion), 24 h and 48 h), was determined by lysing the cells in 0.1% Triton X-100 and plating on LB-agar as described in Materials and Methods. Results are expressed as bacterial load/ 2x10^5^ cells. Inset shows the RT-PCR analysis to confirm the gene knock down. Total RNA was isolated from cells and the expression of Rab14 was assessed by qRT-PCR analysis. Values were normalized GAPDH.
(TIF)

S6 Fig LC3 colocalizes with UCV during intracellular infection of BEC. BEC cells were transfected with GFP-Rab35 for 24 h followed by infection with UPEC. At 24 h post infection the cells were fixed, stained for LC3. The colocalization of Rab35 with LC3 was analyzed by confocal microscopy. The experiment was repeated three times with similar results and a representative Fig is shown. DAPI {blue, host nuclei (N) or bacteria (UPEC)}, Rab35 (green), and LC3 (red). Zoom panel shows the magnified image of one of the UCV’s (marked by arrows in the merged image). The graph shows the quantification of the fluorescence intensity along the white line shown in zoomed image. At least 100 UCVs were counted for each experiment. Experiments were repeated three times with similar results and a representative Fig is shown.
(TIF)

S1 Table Sequences of primers used in the study. (XLSX)
