# Supplementary figures and images for: Intracellular Uropathogenic E. coli Exploits Host Rab35 for Iron Acquisition and Survival within Urinary Bladder Cells
Source: PLoS Pathog. 2015 Aug 6;11(8):e1005083. doi: 10.1371/journal.ppat.1005083 (PMC4527590; doi:10.1371/journal.ppat.1005083)

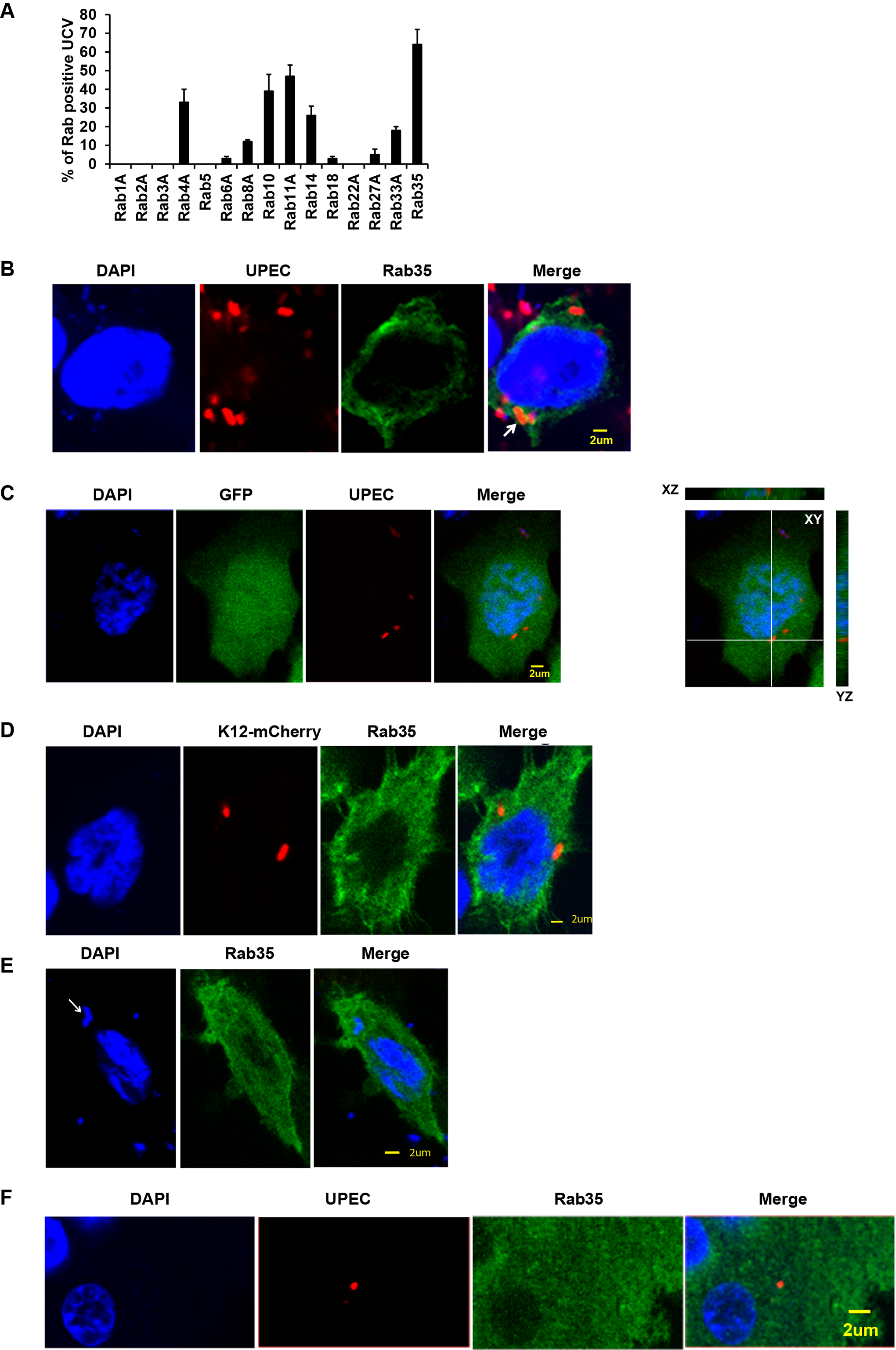

Supplement: S1 Fig — BEC cells were transfected with RFP/GFP tagged RabGTPases. At 24 h post transfection the cells were infected {with RFP/GFP-UPEC (MOI 500)} and the localization of Rab GTPases with UPEC-containing vacuole (UCV) was analyzed at 24 h post-infection by confocal microscopy. Number of RFP/GFP positive UCV was divided by the total number of UCVs and is represented in the graph as % of Rab positive UCV. B. Colocalization of Rab35 with UPEC. BEC cells were transfected with GFP-Rab35. At 24 h post transfection the cells were infected with RFP-UPEC (MOI 500) and the colocalization of UPEC with Rab35 (indicated by arrow in the merged panel) was analyzed at 24 h post-infection by confocal microscopy. DAPI (blue), UPEC (red), Rab35 (green). C. GFP does not localize to UCV. BEC cells overexpressing GFP were infected with RFP-UPEC (MOI 500) for 24 h and analyzed by confocal microscopy. DAPI (blue), GFP (green), and UPEC (red). Also shown at the right side are the orthogonal sections of intracellular bacteria in XZ and YZ plane. White lines represent regions in which XYZ sections were taken. Scale bar denotes 2μm. D. Type1-pili expressing E. coli (K12) do not recruit Rab35. BEC cells overexpressing Rab35-GFP were infected with mCherry-K12 (MOI 500) for 24 h and analyzed by confocal microscopy. DAPI (blue), Rab35 (green), and mCherry-K12 (red). E. Heat-killed UPEC does not recruit Rab35. BEC cells overexpressing Rab35-GFP were infected with heat-killed UPEC (MOI 500) for 24 h and analyzed by confocal microscopy. DAPI (blue, host nuclei or bacteria), and Rab35 (green). Arrows in DAPI panel indicate heat killed UPEC. Experiments were repeated three times with similar results. Representative images are shown. F. UPEC infected mouse bladder sections showing intracellular UPEC that are negative for Rab35. C57BL/6 mice were infected transurethrally with UPEC (UTI89 strain). Mouse bladders were removed at 2 weeks post infection and the tissue sections were processed for immunofluorescence. [file ppat.1005083.s001.tif]

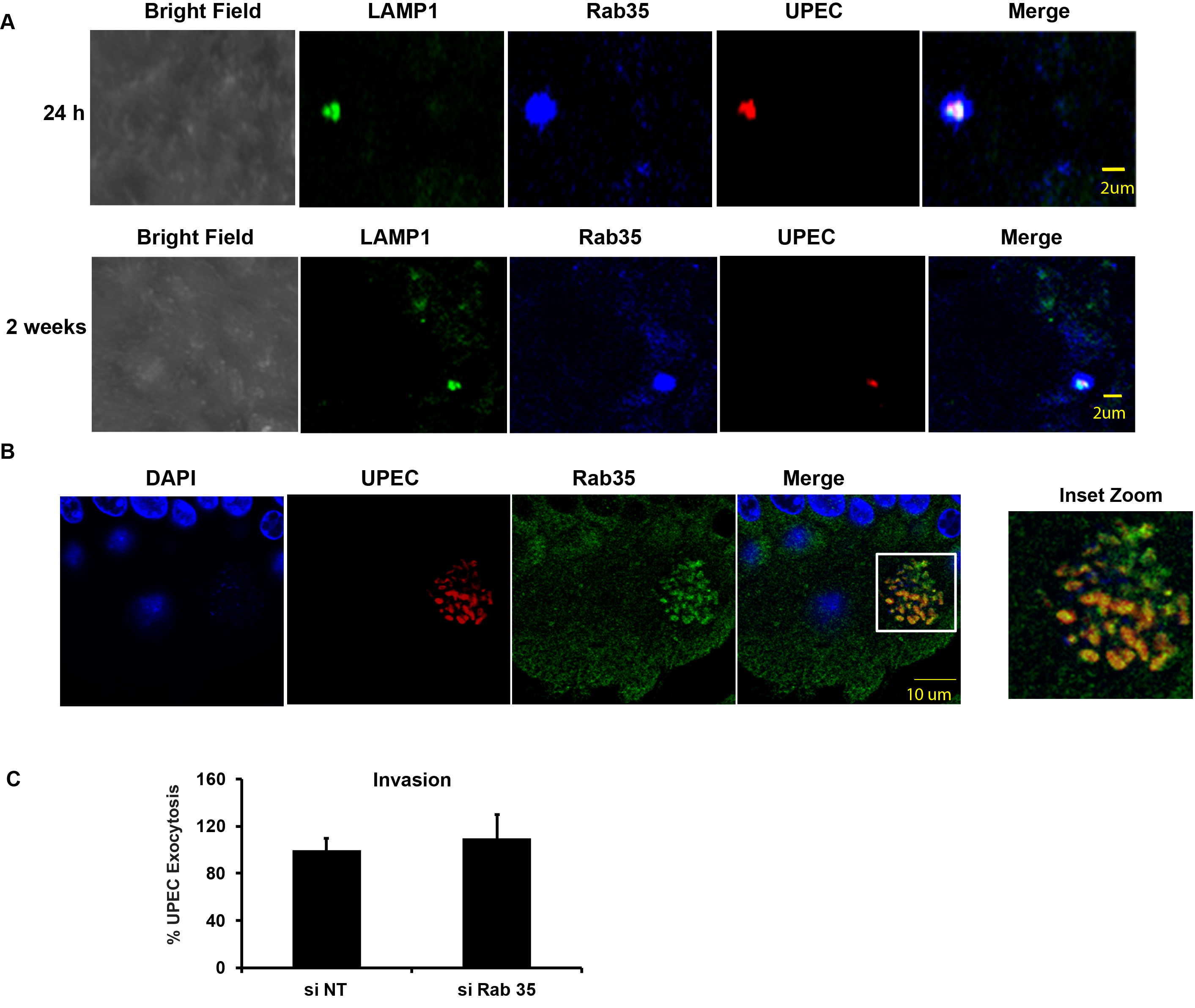

Supplement: S2 Fig — C57BL/6 mice were infected transurethrally with UPEC (UTI89 strain). Mouse bladders were removed 24 h and 2 weeks post infection and the tissue sections were processed for immunofluorescence. Rab35 (blue), UPEC (red) and LAMP1 (green). B. Rab35 associates with IBC forms of UPEC in mouse bladder sections. C57BL/6 mice were infected transurethrally with UPEC (UTI89 strain). Mouse bladders were removed 6 h post infection and the tissue sections were processed for immunofluorescence. Rab35 (green), UPEC (red) and DAPI (blue). n = 4 sections/mouse bladder, n = 3 mice per experiment. C. Rab35 silencing does not enhance the efflux rate of UPEC from BEC-5637 at 4 h post-infection. BEC-5637 cells were transfected with 100nM each of si Rab35 or non-targeting siRNA (si NT). 48 h following knockdown, the cells were infected with UPEC at MOI 500. After gentamycin (100μg/ml) treatment, cells were washed in left in fresh culture medium containing 100mM methyl-D-mannopyranoside. At 4 h post infection, the culture medium was collected and plated for CFU counts as described in Materials and Methods. Results are expressed % exocytosis relative to siNT cells. Values shown represent mean ± standard deviation of results of three independent experiments. (TIF) [file ppat.1005083.s002.tif]

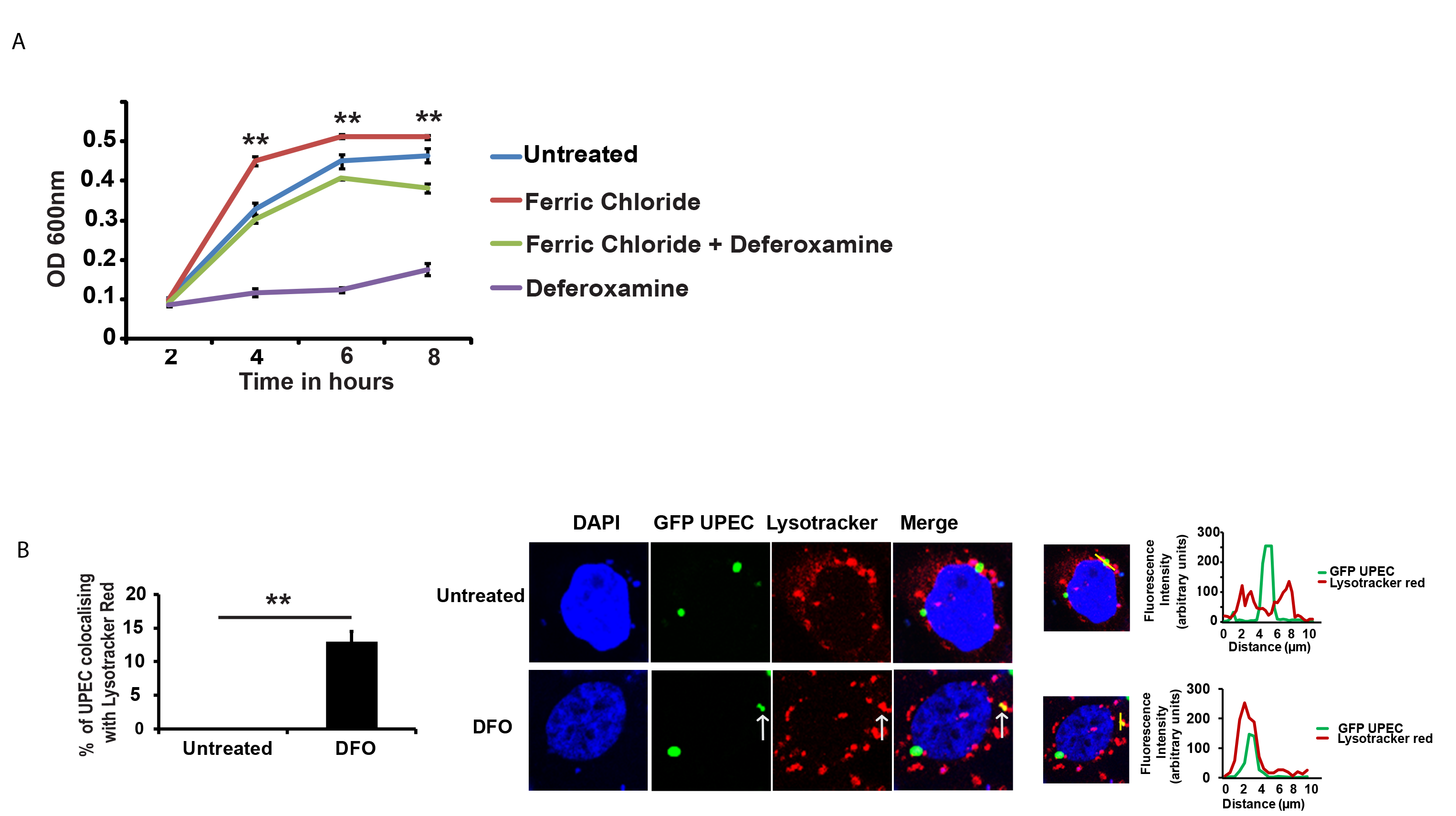

Supplement: S3 Fig — A. UPEC grown in cell-free system (LB media) was supplemented with iron (ferric chloride) or iron chelator deferoxamine for various time points. OD600 was measured at the corresponding time points and plotted as a measure of the UPEC growth. ** represents p<0.01, Values shown represent mean ± standard deviation of results of three independent experiments. B. BEC cells were maintained in serum-free conditions 4 h prior to infection and were subsequently left in serum free medium for the rest of the duration of the experiment. Cells were infected with UPEC at MOI 500 for 2 h. Following infection, and gentamycin treatment, the cells were washed and left in RPMI with gentamycin (10μg/ml) and either left untreated or treated with 100μg/ml Deferoxamine (DFO). Twenty four h later, the cells were stained with LysoTracker Red for 2 h. Subsequently the cells were fixed and analyzed for colocalization between UPEC and LysoTracker Red by confocal microscopy. Graph (left panel) is depicted as % of bacteria co-localizing with LysoTracker Red. At least 100 bacteria were counted. ** represents p< 0.01, Values shown represent mean ± standard deviation. A representative image is shown. Colocalization between UPEC and Lysotracker red is indicated by arrow in the merged image. The graph (right panel) shows the quantification of the fluorescence intensity along the white line. (TIF) [file ppat.1005083.s003.tif]

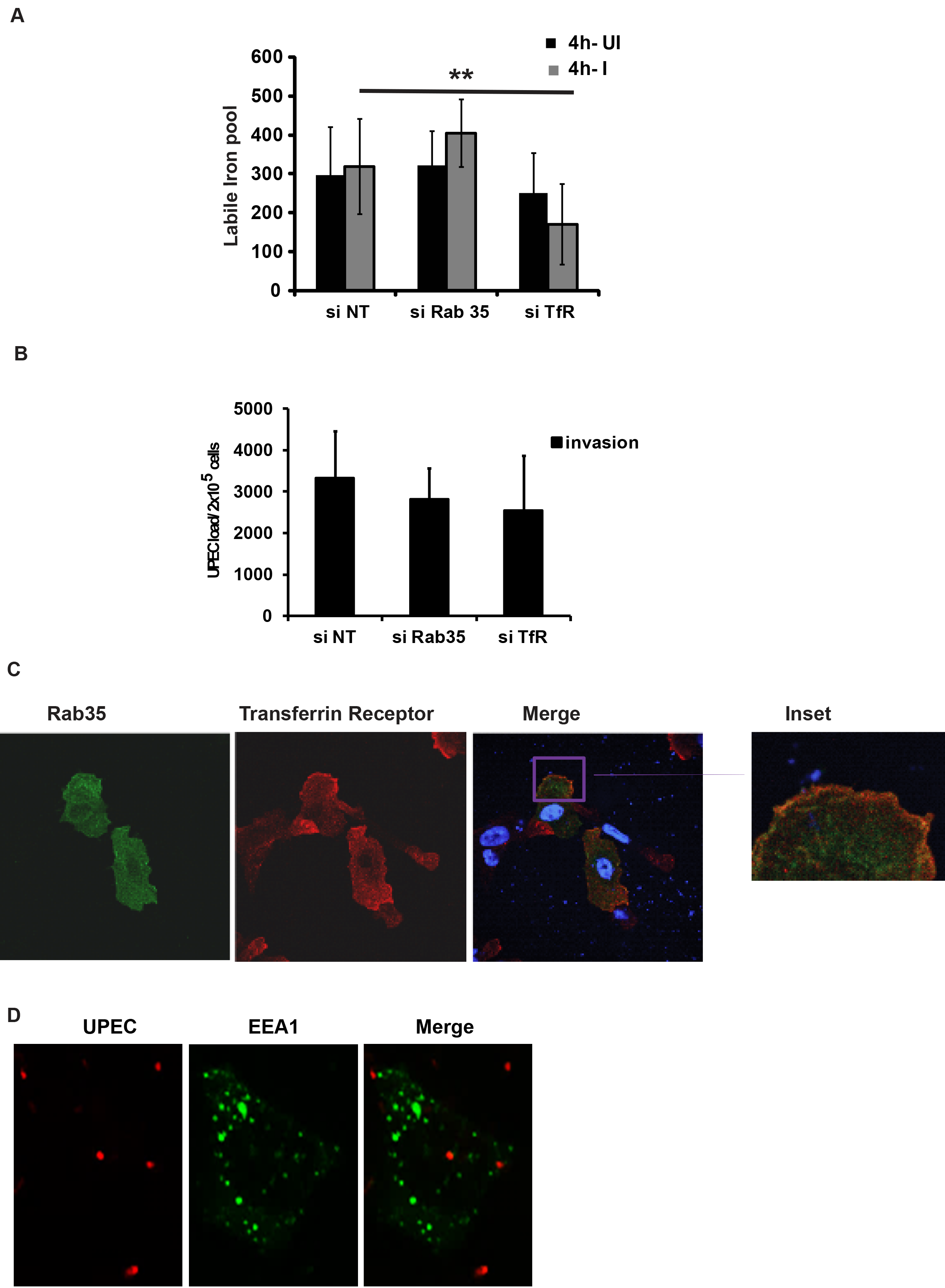

Supplement: S4 Fig — BEC cells transfected with si NT, si Rab35 or si TfR were left either uninfected or infected with UPEC. Intracellular iron levels at 4 h post infection were determined by calcein-AM fluorescence as described in materials and methods. Data represent mean ± standard deviation of results of three independent experiments **represents p<0.01. si = siRNA, si NT = negative control siRNA. B. UPEC load does not change in Rab35 or TfR knockdown cells at 4 h post-infection. BEC cells were transfected with si TfR, si Rab35 or siNT for 48 h followed by infection with UPEC for 4 h. Intracellular bacterial load was determined as described before. Results are expressed as bacterial load/ 2x10^5^ cells. C. Rab35 colocalizes with Transferrin receptor at the cell surface during UPEC infection. BEC cells were transfected with GFP-Rab35 for 24 h followed by UPEC infection. At 24 h post infection the cells were fixed and stained for either cell surface Transferrin Receptor. The colocalization of Rab35 with cell surface Transferrin Receptor was analyzed by confocal microscopy. D. UCV does not colocalize with EEA1. BEC cells were transfected with GFP-EEA1 for 24 h followed by infection with RFP-UPEC. At 24 h post infection the cells were fixed and analyzed by confocal microscopy. The experiment was repeated three times with similar results and a representative Fig is shown. EEA1 (green), and UPEC (red). The experiment was repeated three times with similar results and a representative Fig is shown. (TIF) [file ppat.1005083.s004.tif]

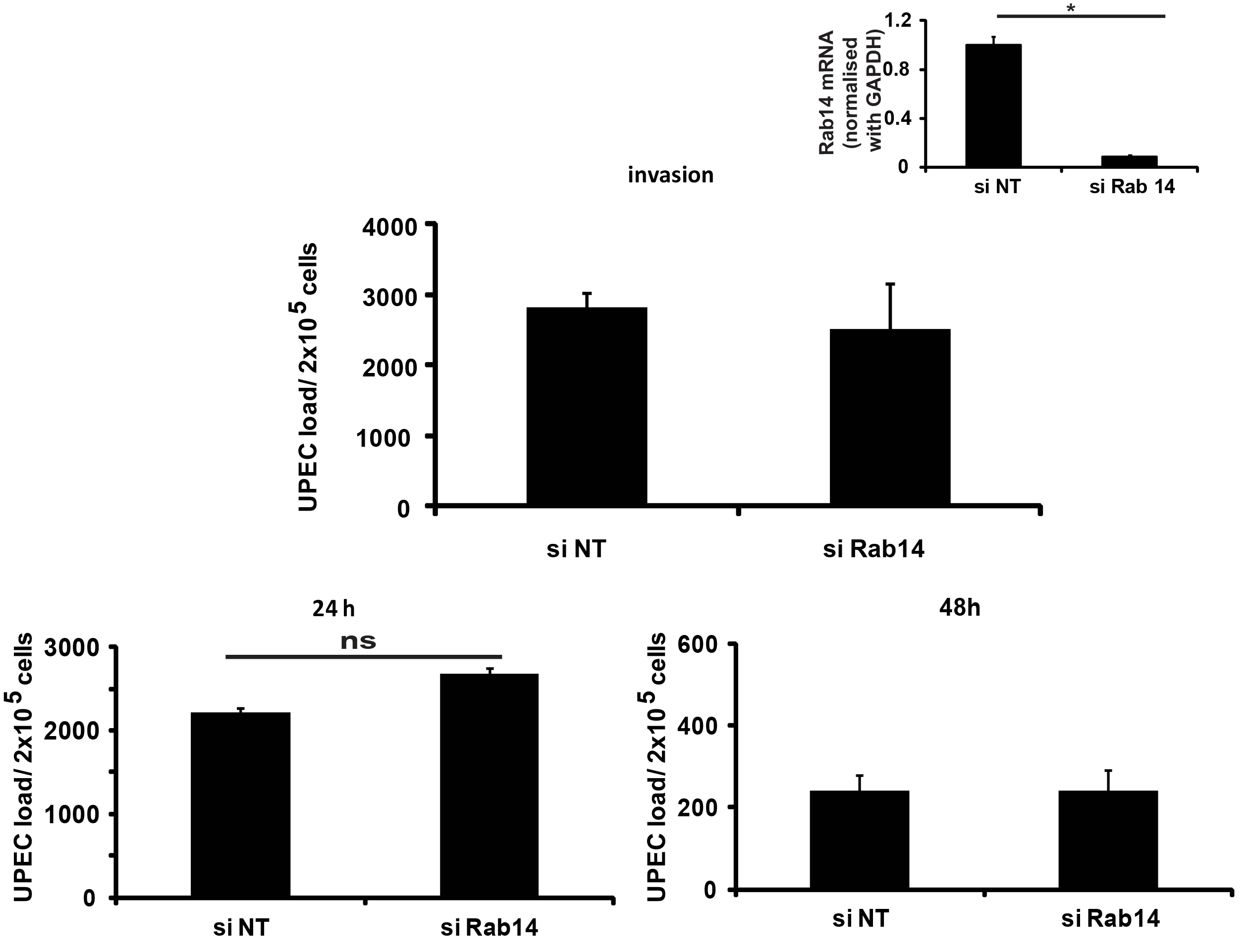

Supplement: S5 Fig — BEC5637 cells were transfected with 100nM each of si Rab14 or si NT. 48h following knockdown, the cells were infected with UPEC at MOI 500. Intracellular bacterial load at different time points {4 h (invasion), 24 h and 48 h), was determined by lysing the cells in 0.1% Triton X-100 and plating on LB-agar as described in Materials and Methods. Results are expressed as bacterial load/ 2x10^5^ cells. Inset shows the RT-PCR analysis to confirm the gene knock down. Total RNA was isolated from cells and the expression of Rab14 was assessed by qRT-PCR analysis. Values were normalized GAPDH. (TIF) [file ppat.1005083.s005.tif]

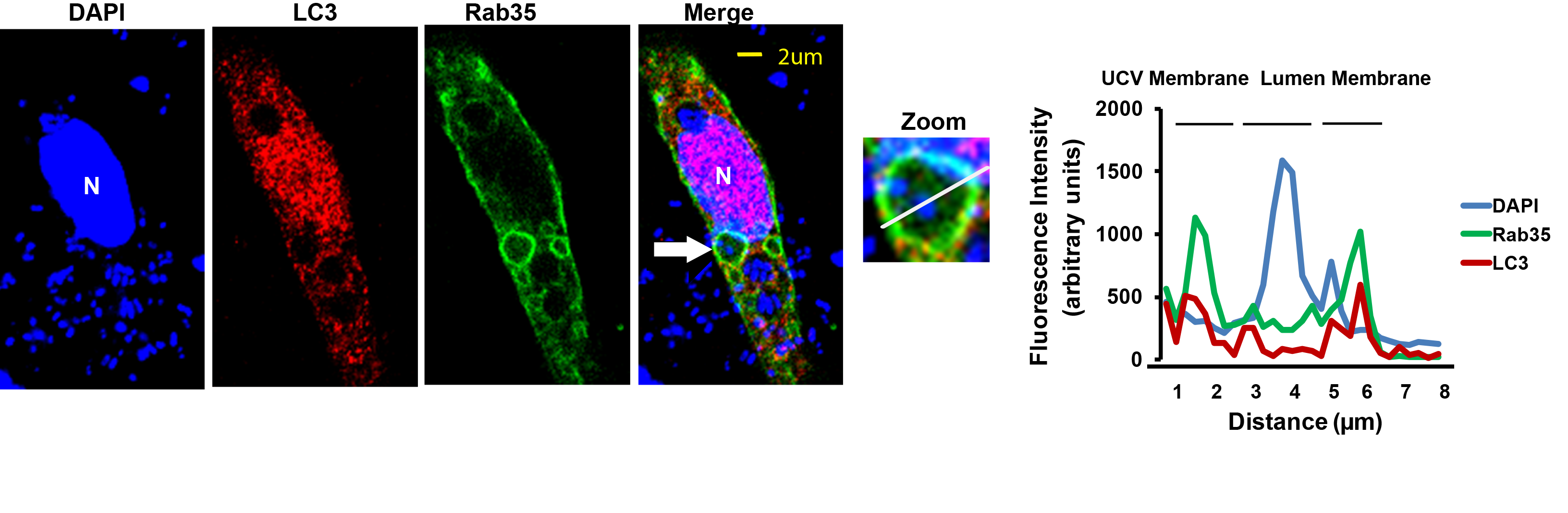

Supplement: S6 Fig — BEC cells were transfected with GFP-Rab35 for 24 h followed by infection with UPEC. At 24 h post infection the cells were fixed, stained for LC3. The colocalization of Rab35 with LC3 was analyzed by confocal microscopy. The experiment was repeated three times with similar results and a representative Fig is shown. DAPI {blue, host nuclei (N) or bacteria (UPEC)}, Rab35 (green), and LC3 (red). Zoom panel shows the magnified image of one of the UCV’s (marked by arrows in the merged image). The graph shows the quantification of the fluorescence intensity along the white line shown in zoomed image. At least 100 UCVs were counted for each experiment. Experiments were repeated three times with similar results and a representative Fig is shown. (TIF) [file ppat.1005083.s006.tif]
